# Generation of genetically modified mice using SpCas9-NG engineered nuclease

**DOI:** 10.1038/s41598-019-49394-5

**Published:** 2019-09-09

**Authors:** Wataru Fujii, Haruka Ito, Takuya Kanke, Arisa Ikeda, Koji Sugiura, Kunihiko Naito

**Affiliations:** Department of Animal Resource Sciences, Graduate School of Agricultural and Life Sciences, The University of Tokyo, 1-1-1 Yayoi, Bunkyo-ku, Tokyo 113-8657 Japan

**Keywords:** Genetic engineering, Transgenic organisms

## Abstract

Although genetically modified mice can be generated with high efficiency by using CRISPR/Cas9-mediated genome editing in mouse zygotes, only the loci with a protospacer-adjacent motif (PAM) sequence are targetable. The present study investigated the usability of engineered *Streptococcus pyogenes* Cas9 (SpCas9-NG) in mouse zygotes. In addition to the 5′-NGG sequence, SpCas9-NG recognized the 5′-NGA, 5′-NGC and 5′-NGT sequences in mouse zygotes as PAMs that were appropriate for the generation of knockout mice. Moreover, SpCas9-NG-mediated genome editing enabled the generation of knock-in mice untargetable by the conventional SpCas9 in mouse zygotes. These results suggest that SpCas9-NG-mediated genome editing in zygotes is available for the generation of knockout and knock-in mice at the locus corresponding to NGN-PAM.

## Introduction

The Clustered Regularly Interspaced Short Palindromic Repeat (CRISPR)/Cas9 system, which consists of Cas9 endonuclease and a single guide-RNA (gRNA)^1,2^, is a widely utilized engineered endonuclease, and CRISPR/Cas9-mediated genome editing in mammalian zygotes is utilized for the generation of genetically modified animals^2–8^. The Cas9-gRNA complex recognizes a specific DNA sequence which consists of a base-pairing sequence to the spacer region of the gRNA and a protospacer adjacent motif (PAM), then induces a DNA double-strand break (DSB) in the target site. The repair of a DSB by error-prone non-homologous end-joining (NHEJ) sometimes forms insertion/deletion (indel) mutations, thereby disrupting the target gene or genome information^7,8^. Besides, DSB stimulates homology-directed repair (HDR), and thus knock-in via HDR is achieved by introducing exogenous donor DNA as a repair template together with the Cas9 and gRNA.

*Streptococcus pyogenes* (Sp)-derived Cas9 (SpCas9) is commonly used as a conventional CRISPR/Cas9 system. SpCas9 requires 5′-NGG as its PAM sequence, and consequently, the targetable locus is restricted^9,10^. Alternatively, other prokaryote-derived orthologous Cas9 endonucleases and Cpf1 (also known as Cas12a), which recognize different sequences as PAMs, are available for genome editing in mammalian cells including zygotes^11–19^. Although these systems contribute to the expansion of targetable loci, the requirement of specific polynucleotide sequences as PAMs still restricts the designable target loci for genome editing. It was reported that an orthologous Cas9 from *Streptococcus canis* can recognize and cut NNG-PAM-bearing target site in mammalian culture cells, but the availability to mammalian zygotes is not investigated^20^.

It has been reported that the protein engineering of Cas9 endonuclease enhances functions such as the requirement of a PAM sequence^21–23^, the accuracy of target recognition^24–28^ or endonuclease activities^29–31^. The xCas9^22^ and SpCas9-NG^23^ are engineered Cas9 containing independent 7 amino acid substitutions of the wildtype SpCas9 and require a 5′-NGN sequence as the PAM. It was reported that SpCas9-NG more efficiently recognize and cleavage the target site bearing NGH-PAM compared with xCas9 in *in vitro* assay^23^ and SpCas9-NG induced NHEJ-mediated indels or nucleotide substitution via a fused-deaminase domain at the target loci corresponding to NGN-PAM in mammalian culture cell and plants^23,32,33^. It was reported that SpCas9-NG increases the targeting range of SpCas9 in the human coding sequence^23^, therefore the utilization of SpCas9-NG in mammalian zygotes is expected to expand the versatility of target designs for the generation of genetically modified animals. However, previous studies have suggested that SpCas9-NG reduces the efficiency of target mutagenesis compared with wildtype SpCas9 at NGG-PAM^23^. Thus, it is unclear whether SpCas9-NG could be used in place of the conventional SpCas9 for genome editing in zygotes.

In the present study, we evaluated the efficiency of SpCas9-NG-mediated genome-editing at endogenous target sites bearing NGN-PAM in mouse zygotes. Moreover, we attempted to generate knockout and knock-in mice using SpCas9-NG.

## Results

We previously established a Cas9 expression construct optimized for mammalian zygotes. This construct-derived Cas9 mRNA has shown highly efficient target mutagenesis at various loci in mouse zygotes^8,18,19,31,34,35^, and we therefore used this plasmid vector as a template when reconstructing SpCas9-NG in the present study. Western blot analysis showed that SpCas9-NG expressed as well as the wildtype Cas9 in HEK293 cells (Supplementary Fig. 2).

By using this construct, we evaluated the efficiency of SpCas9-NG-mediated target mutagenesis in mouse embryos. We designed 9 gRNAs at a tyrosinase locus; they corresponded to the 5′-NGG, 5′-NGA, 5′-NGT and 5′-NGC sequences as PAMs (Supplementary Fig. 3A,B). Each gRNA was microinjected with SpCas9-NG mRNA into C57BL/6NCr-derived zygotes, and then blastocyst-stage embryos were subjected to the PCR-directed Sanger-sequencing, and each of the obtained chromatogram data was observed. As a result, the target sequences of NGA-, NGC- and NGT-PAM contained mutagenized sequence in almost all of the blastocyst (97.7%, 94.2% and 93.2%, respectively) in addition to the target sequence of NGG-PAM (98.1%) by using SpCas9-NG in contrast to the wildtype SpCas9, which generated only 16.7% mutants in NGA-PAM and 6.3% mutant in NGT-PAM (Supplementary Figs 3B,C and 4). The TIDE analysis^35^ also suggested that SpCas9-NG showed highly efficient scores in the target sequence of NGN-PAM in each blastocyst while the rates of mutagenic efficiencies in NGA-PAM and NGT-PAM by wildtype-SpCas9 were limited (2.2 and 2.6%, respectively) (Supplementary Fig. 3B,D). These results suggested that SpCas9-NG could recognize the 5′-NGN sequences as a PAM and functioned efficiently as an engineered endonuclease in mouse zygotes.

**Figure 1:**
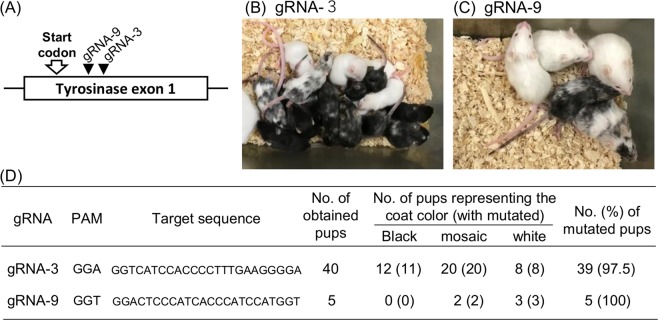
Generation of Tyrosinase knockout mice using SpCas9-NG. (**A**) Schematics of the target loci for generation of tyrosinase knockout mice. (**B**,**C**) The coat colors of obtained pups generated by using gRNA-3 (**B**) and gRNA-9 (**C**). (**D**) The incidence of the represented coat color and mutagenic efficiencies of the obtained pups.

Next, we attempted to generate knockout mice using SpCas9-NG. Cas9 mRNA and gRNA-3 (5′-NGA as PAM; Fig. 1A and Supplementary Fig. 3) were injected into the C57BL/6NCr zygotes and the embryos were transferred to recipients, successfully yielding 40 offspring. Tail-tip-derived genomic DNA indicated that 39 of 40 F0 pups showed induced mutations at the target loci (Fig. 1D and Supplementary Fig. 5). The coat of 28 of 40 pups consisted of completely-white or black-white mosaic hair, suggesting tyrosinase deficiency (Fig. 1B,D). With the same efficiency as gRNA-3, gRNA-9 (5′-NGT as PAM) could induce the mutation efficiently at the target locus (5/5 pups), and the pups exhibited the tyrosinase deficiency (Fig. 1C,D and Supplementary Fig. 6). These results suggested that SpCas9-NG is available for the efficient generation of knockout mice via zygotes.

**Figure 2:**
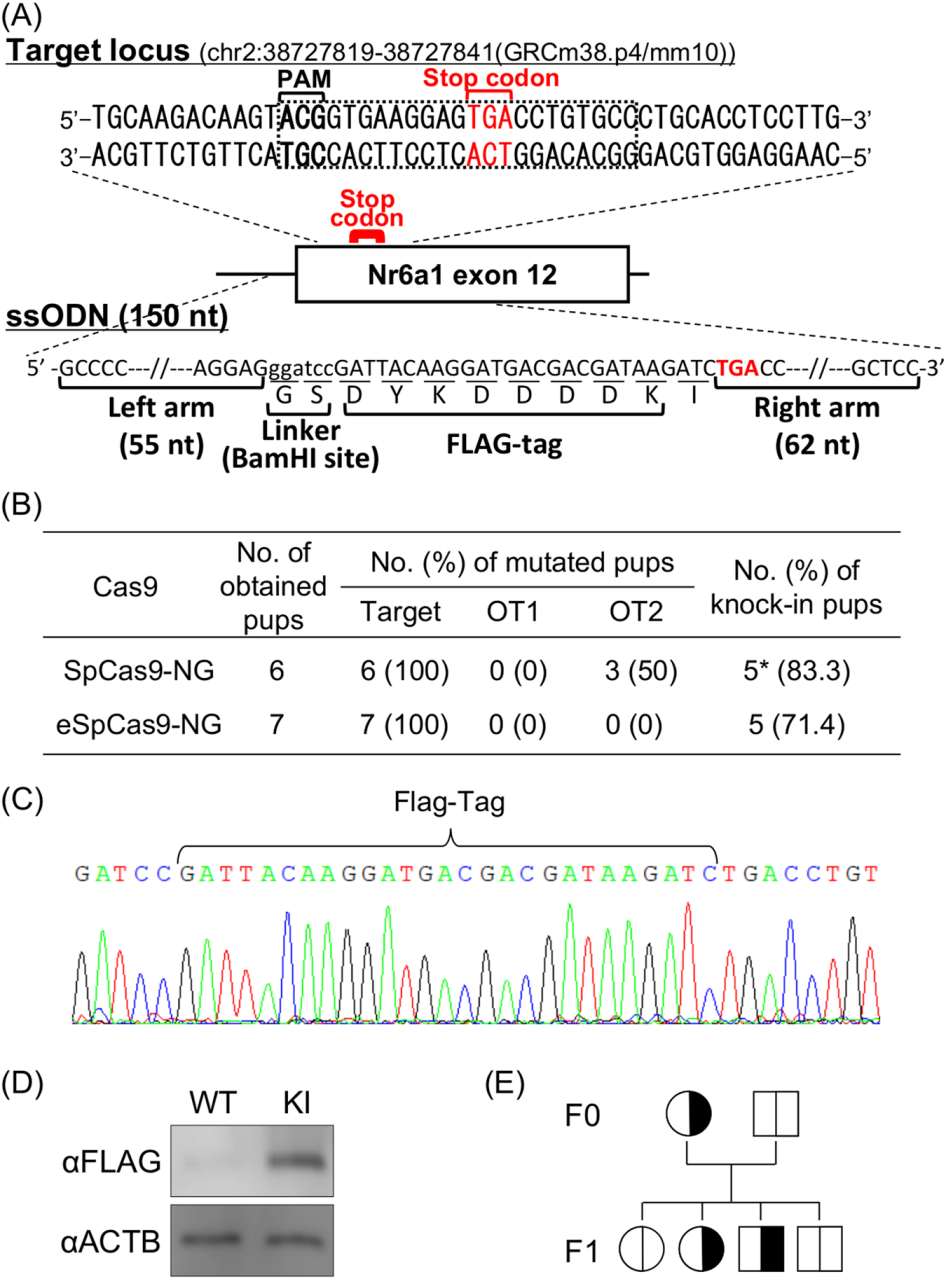
Generation of Nr6a1-Flag knock-in mice using SpCas9-NG and eSpCas9-NG. (**A**) Schematic illustration of the Nr6a1 gene structures, sequences around the target locus (upper), and ssODN template (lower). Black-dot box on the sequence around the target locus indicates the PAM and the sequence recognized by the Cas9-gRNA complex. The target sequence contains the stop codon of Nr6a1. The DNA sequence of ssODN is shown in Supplementary Table 1. (**B**) Mutagenic efficiencies at on-target and off-targets (as shown OT1 and OT2), and knock-in efficiencies of the obtained pups. The locus information is shown in Supplementary Table 2. *Two pups have a nucleotide substitution between Flag-tag encoding sequence and stop codon (shown in Supplementary Fig. 9A). (**C**) Waveform sequence data of the target locus of an obtained pup (see also Supplementary Figs 8 and 9). (**D**) Immunoblotting of Flag-tag (FLAG) and β-Actin (ACTB) using the protein extracted from the testes of wildtype and the knock-in males. (**E**) A pedigree obtained from Nr6a1-Flag knock-in F0 female generated by using SpCas9-NG. The knock-in alleles are shown by the black color, transmitting to the male and female pups (see also Supplementary Fig. 12).

We next examined whether SpCas9-NG-mediated genome editing in zygotes could be utilized for the generation of knock-in mice. In the case of genome editing-mediated knock-in, it is preferable to design gRNA overlapping with the knock-in site in order to avoid retargeting of the knock-in allele^36^. Nuclear receptor subfamily 6, group A, member 1 (Nr6a1) is known as an orphan nuclear receptor which is expressed in a variety of tissues, including testis tissue, in adult mice^37,38^. Knock-in of the epitope-tag into the NR6A1 c-terminal is a suitable experiment for proof-of-principle because there is no canonical PAM (5′-NGG) at the 3′ sides of the stop codon in the plus- and minus-strand sequence (Fig. 2A), which is required for the design of a gRNA overlapping with the knock-in site. Thus, a gRNA (5′-NGT as PAM) was designed, and was microinjected together with SpCas9-NG mRNA and a single strand oligodeoxynucleotide (ssODN)-encoding Flag-tag sequence and homology arms (Fig. 2A). After embryo transfer, 6 pups were successfully obtained. The sequencing of the tail-tip derived-genome DNA revealed that mutagenesis took place in all of the pups (Supplementary Fig. 8A) and 5 of the 6 had the Flag-tag knock-in allele (Fig. 2B,C and Supplementary Figs 7A and 9A). Flag-tagged allele-derived Nr6a1 mRNA was expressed in a knock-in male testis, and Flag-tagged Nr6a1 protein expression was detected by western blot analysis (Fig. 2D and Supplementary Fig. 11). A knock-in female pup was mated with a wildtype male and the obtained offspring revealed that the knock-in allele was inherited normally in the next generation (Fig. 2E and Supplementary Fig. 12). These results suggested that SpCas9-NG-mediated genome editing in zygotes could be utilized for knock-in mouse generation. Meanwhile, 3 pups had unexpected mutations at one of the two examined predicted off-target locus, OT2 (Fig. 2B and Supplementary Fig. 10). Accordingly, we investigated in mouse zygotes the efficacy of the eSpCas9-NG variant—which is SpCas9-NG with the high-fidelity eSpCas9 variant—thereby reducing the off-target risk in somatic culture cells^23^. Although all 7 of the resulting pups had the target modification (Supplementary Fig. 8B) and 5 of them had the knock-in allele (Supplementary Figs 7B and 9B), no mutation was found in OT2 locus of any of the pups (Fig. 2B). These results suggested that eSpCas9-NG would have the potential to be available for the efficient and accurate genome editing in mouse zygotes as well as the previous study by somatic culture cells^23^.

## Discussion

The present study investigated whether SpCas9-NG could be used for genome editing of NG-PAM loci via zygotes, and the results showed that both knockout and knock-in mice could be successfully generated using this approach. To date, the target design for genome editing in zygotes has been restricted to the locus where the specific polynucleotide sequence is present. Since SpCas9-NG requires only mononucleotide “G” as a PAM sequence, it expands the range of targetable loci to include loci that could not be achieved by wildtype SpCas9 and orthologous Cas9 in mammalian zygotes. Such expansion is particularly conducive to the production of knock-in animals. Because the Cas9-gRNA injected into the 1-cell-stage egg maintains its activity for a while during preimplantation development, it may be possible to induce unexpected NHEJ-mediated indels by retargeting of the Cas9-gRNA even if the knock-in is achieved at an early stage. Thus, it is necessary to design a gRNA to overlap the knock-in position so that the post-knock-in sequence is not a target for gRNA^35^. For precise modification of the restricted position, such as nucleotide substitution or insertion of a reporter gene or epitope-tag, a specific gRNA must be designed from limited alternatives. Thus, by increasing the designable loci of gRNA, the SpCas9-NG system is expected to expand the feasibility of such applications.

In contrast, it is concerned that the shortening of the PAM sequence also increases the number of potential off-target positions. In the previous study, off-target mutations by SpCas9-NG have been avoided by the use of the high fidelity eSpCas9 variant^23^, and the present study in mouse zygotes also supported it while it is preliminary. In addition to the eSpCas9 variant, it has been reported that the utilization of other high fidelity Cas9 variants^24–28^, shortening of the protospacer and engineering of a gRNA scaffold structure^39,40^ are all effective means of improving the target recognition of Cas9-gRNA. It is possible that these improvements would contribute to a reduction of the off-target risk of SpCas9-NG in zygotes.

In this study, we found the presence of mutated alleles was observed in almost all obtained offspring, and also observed the transmission of knock-in alleles to the next generation. In contrast, some pups exhibit a mosaic or black coat color as a phenotype while the mutant allele was detected by genotyping, suggesting that the wildtype allele remains as heterozygous mutation at a certain rate in mutated pups. As well, the results of TIDE assay showed various rates of the efficiencies because of containing an intact allele in some of the genetically-modified blastocysts (Supplementary Fig. 3D). In our previous studies on tyrosinase knockout using SpCas9, ST1-Cas9 or Cj-Cas9, almost all of the pups showed bi-allelic target mutagenesis and presented a completely white coat^18,19,34^. SpCas9-NG has been reported to have lower mutagenic activity compared to wildtype SpCas9 at NGG-PAM^23^, which could have affected the present results in zygotes. Additional improvements to the mutagenic activity of SpCas9-NG are expected, and should further expand the feasibility of efficient genome editing in zygotes.

## Material and Methods

### Ethics statement

All animal care and experiments conformed to the Guidelines for Animal Experiments of the University of Tokyo and were approved by the Animal Research Committee of the University of Tokyo (P18-093, 27 Sept 2018).

### Construction of Cas9- and gRNA-plasmid DNA

According to the amino acid sequences shown in previous studies^23,24^, SpCas9-NG and its high-fidelity variant, eSpCas9-NG, were constructed by mutation PCR of pCAG-T3-hCAS9-pA plasmid vector (Addgene plasmid 48625)^8^. The vectors were sequenced using a commercial sequencing kit (Applied Biosystems, Foster City, CA, USA) and a DNA sequencer (Applied Biosystems) according to the manufacturer’s instructions. The gRNAs for tyrosinase and Nr6a1 were newly designed or designed according to a previous study^34^, and plasmid vectors coding gRNAs with a T3 promoter for each target were synthesized according to a previous study^8^. These vectors were sequenced as described above. All of the sequence information is provided in Supplementary Fig. 1.

### *In Vitro* transcription of Cas9 mRNA and gRNAs

The *in vitro* synthesis of Cas9 mRNA and gRNA was performed as described previously^8^ using T3 RNA polymerase (New England Biolabs, MA, USA). The RNA transcripts were precipitated with absolute ethanol, washed, and resuspended in RNase-free water. The RNA solutions were stored at −80 °C until use.

### Microinjection and embryo-transfer

The microinjection was performed using a microinjector (Narishige, Tokyo, Japan)-equipped microscope. Approximately 4 pL of RNA solution, containing 100 ng/μL of Cas9 mRNA and 20 ng/μL of gRNA (with 100 ng/μL ssODN as shown in Supplementary Table 1 only for the knock-in experiment), was injected into the cytoplasm of *in vitro* fertilization-derived C57BL/6NCr zygotes^19^. The injected zygotes were cultured in KSOMaa-BSA for 1–2 h, then transferred into the oviducts of 0.5 days post-coitum pseudopregnant ICR females; alternatively, the zygotes were cultured for 96 h and blastocysts were subjected to genotyping.

### Detection of induced mutations at the on- and off-target sites

A search for a potential off-target locus for NR6A1 gRNA was performed using a CRISPOR Version 4.92 (http://crispor.tefor.net/)^41^. Briefly, an NR6A1 target sequence (5′-GGCACAGGTCACTCCTTCACCGT) was used for the search and the “20bp-NGN or GA(A/T)” was searched as PAM, then the resulting loci (off-targets for 0-1-2-3-4 mismatches were 0 - 0 - 2 - 52 - 518 loci) were adopted as potential off-target loci (Supplementary Table 2), and two of the resulting loci which has 2 mismatches were subjected to the analysis of mutation induction (Supplementary Table 3).

The genome DNA was extracted from the blastocysts or the tail tips of the pups according to our previous report^19^, then subjected to PCR using the primer sets shown in Supplementary Table 1. The PCR amplicons were purified by a FastGene Gel/PCR Extraction Kit (Nippon Genetics, Tokyo) and sequenced as described above. Mutation introduction in chromatogram data was detected as insertion or deletion of nucleotides into the wildtype sequence or as overlapping of multiple waveforms at the target site, as shown in a previous study^8^. Furthermore, each chromatogram data of blastocyst-derived Sanger sequencing was subjected to TIDE assay^34^ to measure the rates of mutant sequence in each blastocyst. TIDE-derived “overall efficiency” and its median of all blastocysts in each group were shown as the efficiency scores (Supplementary Fig. 3B,D).

### Cell culture and DNA transfection

The HEK293 cell culture and DNA transfection were performed according to the previous report^19,42^. The constructed Cas9-expressing vector was transfected into the HEK293 cells by Lipofectamine LTX reagent (Life Technologies, Carlsbad, CA, USA) according to the manufacturer’s protocols. The transfected cells were harvested 48 h after transfection and were subjected to western blot analysis.

### RT-PCR

Total RNA was isolated from testicular cells using TRIzol reagent (Invitrogen, Carlsbad, CA, USA), and first-strand cDNA was produced using ReverTra Ace qPCR RT Master Mix (ToYoBo, Tokyo) according to the manufacturer’s protocol. PCR was performed using the primer sets shown in Supplementary Table 1.

### PCR-restriction fragment-length polymorphism (RFLP)

The genomic PCR- or RT-PCR-produced amplicons were purified as described above and digested with BamHI (TaKaRa, Tokyo), and then the digested materials were measured by agarose-gel electrophoresis. In order to analyze the knock-in DNA sequence, the digested fragments of genomic PCR were extracted and sequenced by using the Nr6a1 reverse primer (Supplementary Table 2).

### Immunoblotting

Western blot analysis was performed according to the process described in our previous report^19^. The harvested culture cells or testicular cells were suspended in Laemmli buffer. The antibodies used were anti-Flag M2 monoclonal antibody (F1804, Sigma, Carlsbad, CA, USA) and anti-β-actin polyclonal antibody (GTX109639, GeneTex, Inc., CA, USA). To visualize the protein-bound antibodies, horseradish peroxidase-conjugated anti-mouse IgG and anti-rabbit IgG (Jackson ImmunoResearch Laboratories, Inc., West Grove, PA, USA) were used for the second layer, respectively. The signals were detected using an Immunostar LD Kit (Wako, Tokyo) and a C-DiGit scanner.

## Supplementary information

Supplementary Informations
