# Supplementary material for: Generation of genetically modified mice using SpCas9-NG engineered nuclease
Source: Sci Rep. 2019 Sep 9;9:12878. doi: 10.1038/s41598-019-49394-5 (PMC6733909; doi:10.1038/s41598-019-49394-5)
Supplement: Supplementary file 1 — Supplementary Informations [file 41598_2019_49394_MOESM1_ESM.pdf]

Generation of genetically modified mice using SpCas9-NG engineered nuclease

Wataru Fujii, Haruka Ito, Takuya Kanke, Arisa Ikeda, Koji Sugiura, Kunihiko Naito

Supplementary Information

Supplementary Figure 1. Sequences of SpCas9-NG, eSpCas9-NG and gRNAs.
Supplementary Figure 2. Confirmation of Cas9 expression by immunoblotting.
Supplementary Figure 3. Schematics of the target loci of each gRNA and summary of the obtained results on embryos.
Supplementary Figure 4. Direct sequencing analysis of tyrosinase-targeted blastocysts.
Supplementary Figure 5. Direct sequencing analysis of tyrosinase-targeted pups using gRNA-3.
Supplementary Figure 6. Direct sequencing analysis of tyrosinase-targeted pups using gRNA-9.
Supplementary Figure 7. Nr6a1-Flag knock-in F0 pups detected by PCR-RFLP.
Supplementary Figure 8. Direct sequencing analysis of Nr6a1-targeted pups.
Supplementary Figure 9. Flag-tag knock-in detected by sequencing of digested PCR fragments.
Supplementary Figure 10. Off-target mutations of Nr6a1-Flag F0 pups.
Supplementary Figure 11. RT-PCR of Nr6a1-Flag knock-in mice.
Supplementary Figure 12. PCR-RFLP of Nr-6a1-Flag F1 pups.
Supplementary Figure 13. Full-length Western blot images.

Supplementary Table 1 Primer sets for genomic PCR and RT-PCR.
Supplementary Table 2 List of potential off-target loci for Nr6a1 on-target sequence.
Supplementary Table 3 Nr6a1 on-target locus and examined off-target loci.

Supplementary Figure 1. Sequences of SpCas9-NG, eSpCas9-NG and gRNAs.

>DNA sequence of SpCas9-NG vector (from T3 promoter to SphI site)

GAAATTAACCCTCACTAAAGGGAACAAAAGCTGGAGCTCCACCGCGGTGGCGGCCGCTCTAGCCCGGGCGGA
TCCACCATGGATTACAAGGATGACGACGATAAGATCATGGCCCCAAAGAAGAAGCGGAAGGTCGGTATCCAC
GGAGTCCCAGCAGCCGACAAGAAGTACTCCATTGGGCTCGATATCGGCACAAACAGCGTCGGCTGGGCCGTC
ATTACGGACGAGTACAAGGTGCCGAGCAAAAAATTCAAAGTTCTGGGCAATACCGATCGCCACAGCATAAAG
AAGAACCTCATTGGCGCCCTCCTGTTCGACTCCGGGGAGACGGCCGAAGCCACGCGGCTCAAAAGAACAGCA
CGGCGCAGATATACCCGCAGAAAGAATCGGATCTGCTACCTGCAGGAGATCTTTAGTAATGAGATGGCTAAG
GTGGATGACTCTTTCTTCCATAGGCTGGAGGAGTCCTTTTTGGTGGAGGAGGATAAAAAGCACGAGCGCCAC
CCAATCTTTGGCAATATCGTGGACGAGGTGGCGTACCATGAAAAGTACCCAACCATATATCATCTGAGGAAG
AAGCTTGTAGACAGTACTGATAAGGCTGACTTGCGGTTGATCTATCTCGCGCTGGCGCATATGATCAAATTTC
GGGGACACTTCCTCATCGAGGGGGACCTGAACCCAGACAACAGCGATGTCGACAAACTCTTTATCCAACTGG
TTCAGACTTACAATCAGCTTTTCGAAGAGAACCCGATCAACGCATCCGGAGTTGACGCCAAAGCAATCCTGA
GCGCTAGGCTGTCCAAATCCCGGCGGCTCGAAAACCTCATCGCACAGCTCCCTGGGGAGAAGAAGAACGGCC
TGTTTGGTAATCTTATCGCCCTGTCACTCGGGCTGACCCCCAACTTTAAATCTAACTTCGACCTGGCCGAAGAT
GCCAAGCTTCAACTGAGCAAAGACACCTACGATGATGATCTCGACAATCTGCTGGCCCAGATCGGCGACCAG
TACGCAGACCTTTTTTTGGCGGCAAAGAACCTGTCAGACGCCATTCTGCTGAGTGATATTCTGCGAGTGAACA
CGGAGATCACCAAAGCTCCGCTGAGCGCTAGTATGATCAAGCGCTATGATGAGCACCACCAAGACTTGACTT
TGCTGAAGGCCCTTGTCAGACAGCAACTGCCTGAGAAGTACAAGGAAATTTTCTTCGATCAGTCTAAAAATG
GCTACGCCGGATACATTGACGGCGGAGCAAGCCAGGAGGAATTTTACAAATTTATTAAGCCCATCTTGGAAA
AAATGGACGGCACCGAGGAGCTGCTGGTAAAGCTTAACAGAGAAGATCTGTTGCGCAAACAGCGCACTTTCG
ACAATGGAAGCATCCCCCACCAGATTCACCTGGGCGAACTGCACGCTATCCTCAGGCGGCAAGAGGATTTCT
ACCCCTTTTTGAAAGATAACAGGGAAAAGATTGAGAAAATCCTCACATTTCGGATACCCTACTATGTAGGCCC
CCTCGCCCGGGGAAATTCCAGATTCGCGTGGATGACTCGCAAATCAGAAGAGACCATCACTCCCTGGAACTT
CGAGGAAGTCGTGGATAAGGGGGCCTCTGCCCAGTCCTTCATCGAAAGGATGACTAACTTTGATAAAAATCT
GCCTAACGAAAAGGTGCTTCCTAAACACTCTCTGCTGTACGAGTACTTCACAGTTTATAACGAGCTCACCAAG
GTCAAATACGTCACAGAAGGGATGAGAAAGCCAGCATTCCTGTCTGGAGAGCAGAAGAAAGCTATCGTGGA
CCTCCTCTTCAAGACGAACCGGAAAGTTACCGTGAAACAGCTCAAAGAAGACTATTTCAAAAAGATTGAATG
TTTCGACTCTGTTGAAATCAGCGGAGTGGAGGATCGCTTCAACGCATCCCTGGGAACGTATCACGATCTCCTG
AAAATCATTAAAGACAAGGACTTCCTGGACAATGAGGAGAACGAGGACATTCTTGAGGACATTGTCCTCACC
CTTACGTTGTTTGAAGATAGGGAGATGATTGAAGAACGCTTGAAAACTTACGCTCATCTCTTCGACGACAAAG
TCATGAAACAGCTCAAGAGGCGCCGATATACAGGATGGGGGCGGCTGTCAAGAAAACTGATCAATGGGATCC
GAGACAAGCAGAGTGGAAAGACAATCCTGGATTTTCTTAAGTCCGATGGATTTGCCAACCGGAACTTCATGC
AGTTGATCCATGATGACTCTCTCACCTTTAAGGAGGACATCCAGAAAGCACAAGTTTCTGGCCAGGGGGACA
GTCTTCACGAGCACATCGCTAATCTTGCAGGTAGCCCAGCTATCAAAAAGGGAATACTGCAGACCGTTAAGG
TCGTGGATGAACTCGTCAAAGTAATGGGAAGGCATAAGCCCGAGAATATCGTTATCGAGATGGCCCGAGAGA
ACCAAACTACCCAGAAGGGACAGAAGAACAGTAGGGAAAGGATGAAGAGGATTGAAGAGGGTATAAAAGA
ACTGGGGTCCCAAATCCTTAAGGAACACCCAGTTGAAAACACCCAGCTTCAGAATGAGAAGCTCTACCTGTA
CTACCTGCAGAACGGCAGGGACATGTACGTGGATCAGGAACTGGACATCAATCGGCTCTCCGACTACGACGT
GGATCATATCGTGCCCCAGTCTTTTCTCAAAGATGATTCTATTGATAATAAAGTGTTGACAAGATCCGATAAA
AATAGAGGGAAGAGTGATAACGTCCCCTCAGAAGAAGTTGTCAAGAAAATGAAAAATTATTGGCGGCAGCT

GCTGAACGCCAAACTGATCACACAACGGAAGTTCGATAATCTGACTAAGGCTGAACGAGGTGGCCTGTCTGA
GTTGGATAAAGCCGGCTTCATCAAAAGGCAGCTTGTTGAGACACGCCAGATCACCAAGCACGTGGCCCAAAT
TCTCGATTCACGCATGAACACCAAGTACGATGAAAATGACAAACTGATTCGAGAGGTGAAAGTTATTACTCT
GAAGTCTAAGCTGGTCTCAGATTTCAGAAAGGACTTTCAGTTTTATAAGGTGAGAGAGATCAACAATTACCAC
CATGCGCATGATGCCTACCTGAATGCAGTGGTAGGCACTGCACTTATCAAAAAATATCCCAAGCTTGAATCTG
AATTTGTTTACGGAGACTATAAAGTGTACGATGTTAGGAAAATGATCGCAAAGTCTGAGCAGGAAATAGGCA
AGGCCACCGCTAAGTACTTCTTTTACAGCAATATTATGAATTTTTTCAAGACCGAGATTACACTGGCCAATGG
AGAGATTCGGAAGCGACCACTTATCGAAACAAACGGAGAAACAGGAGAAATCGTGTGGGACAAGGGTAGGG
ATTTCGCGACAGTCCGGAAGGTCCTGTCCATGCCGCAGGTGAACATCGTTAAAAAGACCGAAGTACAGACCG
GAGGCTTCTCCAAGGAAAGTATCAGACCGAAAAGGAACAGCGACAAGCTGATCGCACGCAAAAAAGATTGG
GACCCCAAGAAATACGGCGGATTCGTGTCTCCTACAGTCGCTTACAGTGTACTGGTTGTGGCCAAAGTGGAG
AAAGGGAAGTCTAAAAAACTCAAAAGCGTCAAGGAACTGCTGGGCATCACAATCATGGAGCGATCAAGCTTC
GAAAAAAACCCCATCGACTTTCTCGAGGCGAAAGGATATAAAGAGGTCAAAAAAGACCTCATCATTAAGCTT
CCCAAGTACTCTCTCTTTGAGCTTGAAAACGGCCGGAAACGAATGCTCGCTAGTGCGAGATTCCTGCAGAAA
GGTAACGAGCTGGCACTGCCCTCTAAATACGTTAATTTCTTGTATCTGGCCAGCCACTATGAAAAGCTCAAAG
GGTCTCCCGAAGATAATGAGCAGAAGCAGCTGTTCGTGGAACAACACAAACACTACCTTGATGAGATCATCG
AGCAAATAAGCGAATTCTCCAAAAGAGTGATCCTCGCCGACGCTAACCTCGATAAGGTGCTTTCTGCTTACAA
TAAGCACAGGGATAAGCCCATCAGGGAGCAGGCAGAAAACATTATCCACTTGTTTACTCTGACCAACTTGGG
CGCGCCTAGAGCCTTCAAGTACTTCGACACCACCATAGACAGAAAGGTGTACAGATCTACAAAGGAGGTCCT
GGACGCCACACTGATTCATCAGTCAATTACGGGGCTCTATGAAACAAGAATCGACCTCTCTCAGCTCGGTGGA
GACAGCAGGGCTGACCCCAAGAAGAAGAGGAAGGTGTGAATCGATGGTACCTATGGGCACCAAAGAACCTG
TAAACGTTATCTTTTTAAATTGAATGTGCACAAATAAAAGTTTGGAAAAGAAAAAAAAAAAAAAAAAAAAA
AAAAAAAAAAAAAAAAAAAAAAAAAAAAAAAAAAAAAAAAAAAAAAAAAAAAAAAAAAAAAAAAAAAAA
AAAAACCCTCGAGGTAGCATGC

>Amino acid sequence of SpCas9-NG (L1111R/D1135V/G1218R/E1219F/A1322R/R1335V/T1337R)
MDYKDDDDKIMAPKKKRKVGIHGVPAADKKYSIGLDIGTNSVGWAVITDEYKVPSKKFKVLGNTDRHSIKKNLIG
ALLFDSGETAEATRLKRTARRRYTRRKNRICYLQEIFSNEMAKVDDSFFHRLEESFLVEEDKKHERHPIFGNIVDEV
AYHEKYPTIYHLRKKLVDSTDKADLRLIYLALAHMIKFRGHFLIEGDLNPDNSDVDKLFIQLVQTYNQLFEENPINA
SGVDAKAILSARLSKSRRLENLIAQLPGEKKNGLFGNLIALSLGLTPNFKSNFDLAEDAKLQLSKDTYDDDLDNLL
AQIGDQYADLFLAAKNLSDAILLSDILRVNTEITKAPLSASMIKRYDEHHQDLTLLKALVRQQLPEKYKEIFFDQSK
NGYAGYIDGGASQEEFYKFIKPILEKMDGTEELLVKLNREDLLRKQRTFDNGSIPHQIHLGELHAILRRQEDFYPFL
KDNREKIEKILTFRIPYYVGPLARGNSRFAWMTRKSEETITPWNFEEVVDKGASAQSFIERMTNFDKNLPNEKVLP
KHSLLYEYFTVYNELTKVKYVTEGMRKPAFLSGEQKKAIVDLLFKTNRKVTVKQLKEDYFKKIECFDSVEISGVED
RFNASLGTYHDLLKIIKDKDFLDNEENEDILEDIVLTLTLFEDREMIEERLKTYAHLFDDKVMKQLKRRRYTGWGR
LSRKLINGIRDKQSGKTILDFLKSDGFANRNFMQLIHDDSLTFKEDIQKAQVSGQGDSLHEHIANLAGSPAIKKGILQ
TVKVVDELVKVMGRHKPENIVIEMARENQTTQKGQKNSRERMKRIEEGIKELGSQILKEHPVENTQLQNEKLYLY
YLQNGRDMYVDQELDINRLSDYDVDHIVPQSFLKDDSIDNKVLTRSDKNRGKSDNVPSEEVVKKMKNYWRQLLN
AKLITQRKFDNLTKAERGGLSELDKAGFIKRQLVETRQITKHVAQILDSRMNTKYDENDKLIREVKVITLKSKLVS
DFRKDFQFYKVREINNYHHAHDAYLNAVVGTALIKKYPKLESEFVYGDYKVYDVRKMIAKSEQEIGKATAKYFF
YSNIMNFFKTEITLANGEIRKRPLIETNGETGEIVWDKGRDFATVRKVLSMPQVNIVKKTEVQTGGFSKESIRPKRN
SDKLIARKKDWDPKKYGGFVSPTVAYSVLVVAKVEKGKSKKLKSVKELLGITIMERSSFEKNPIDFLEAKGYKEV

KKDLIIKLPKYSLFELENGRKRMLASARFLQKGNELALPSKYVNFLYLASHYEKLKGSPEDNEQKQLFVEQHKHY
LDEIIEQISEFSKRVILADANLDKVLSAYNKHRDKPIREQAENIIHLFTLTNLGAPRAFKYFDTTIDRKVYRSTKEVLD
ATLIHQSITGLYETRIDLSQLGGDSRADPKKKRKV

>DNA sequence of eSpCas9-NG vector (from T3 promoter to SphI site)
GAAATTAACCCTCACTAAAGGGAACAAAAGCTGGAGCTCCACCGCGGTGGCGGCCGCTCTAGC
CCGGGCGGATCCACCATGGATTACAAGGATGACGACGATAAGATCATGGCCCCAAAGAAGAA
GCGGAAGGTCGGTATCCACGGAGTCCCAGCAGCCGACAAGAAGTACTCCATTGGGCTCGATAT
CGGCACAAACAGCGTCGGCTGGGCCGTCATTACGGACGAGTACAAGGTGCCGAGCAAAAAAT
TCAAAGTTCTGGGCAATACCGATCGCCACAGCATAAAGAAGAACCTCATTGGCGCCCTCCTGT
TCGACTCCGGGGAGACGGCCGAAGCCACGCGGCTCAAAAGAACAGCACGGCGCAGATATACC
CGCAGAAAGAATCGGATCTGCTACCTGCAGGAGATCTTTAGTAATGAGATGGCTAAGGTGGAT
GACTCTTTCTTCCATAGGCTGGAGGAGTCCTTTTTGGTGGAGGAGGATAAAAAGCACGAGCGC
CACCCAATCTTTGGCAATATCGTGGACGAGGTGGCGTACCATGAAAAGTACCCAACCATATAT
CATCTGAGGAAGAAGCTTGTAGACAGTACTGATAAGGCTGACTTGCGGTTGATCTATCTCGCG
CTGGCGCATATGATCAAATTTCGGGGACACTTCCTCATCGAGGGGGACCTGAACCCAGACAAC
AGCGATGTCGACAAACTCTTTATCCAACTGGTTCAGACTTACAATCAGCTTTTCGAAGAGAACC
CGATCAACGCATCCGGAGTTGACGCCAAAGCAATCCTGAGCGCTAGGCTGTCCAAATCCCGGC
GGCTCGAAAACCTCATCGCACAGCTCCCTGGGGAGAAGAAGAACGGCCTGTTTGGTAATCTTA
TCGCCCTGTCACTCGGGCTGACCCCCAACTTTAAATCTAACTTCGACCTGGCCGAAGATGCCAA
GCTTCAACTGAGCAAAGACACCTACGATGATGATCTCGACAATCTGCTGGCCCAGATCGGCGA
CCAGTACGCAGACCTTTTTTTGGCGGCAAAGAACCTGTCAGACGCCATTCTGCTGAGTGATATT
CTGCGAGTGAACACGGAGATCACCAAAGCTCCGCTGAGCGCTAGTATGATCAAGCGCTATGAT
GAGCACCACCAAGACTTGACTTTGCTGAAGGCCCTTGTCAGACAGCAACTGCCTGAGAAGTAC
AAGGAAATTTTCTTCGATCAGTCTAAAAATGGCTACGCCGGATACATTGACGGCGGAGCAAGC
CAGGAGGAATTTTACAAATTTATTAAGCCCATCTTGGAAAAAATGGACGGCACCGAGGAGCTG
CTGGTAAAGCTTAACAGAGAAGATCTGTTGCGCAAACAGCGCACTTTCGACAATGGAAGCATC
CCCCACCAGATTCACCTGGGCGAACTGCACGCTATCCTCAGGCGGCAAGAGGATTTCTACCCCT
TTTTGAAAGATAACAGGGAAAAGATTGAGAAAATCCTCACATTTCGGATACCCTACTATGTAG
GCCCCCTCGCCCGGGGAAATTCCAGATTCGCGTGGATGACTCGCAAATCAGAAGAGACCATCA
CTCCCTGGAACTTCGAGGAAGTCGTGGATAAGGGGGCCTCTGCCCAGTCCTTCATCGAAAGGA
TGACTAACTTTGATAAAAATCTGCCTAACGAAAAGGTGCTTCCTAAACACTCTCTGCTGTACGA
GTACTTCACAGTTTATAACGAGCTCACCAAGGTCAAATACGTCACAGAAGGGATGAGAAAGCC
AGCATTCCTGTCTGGAGAGCAGAAGAAAGCTATCGTGGACCTCCTCTTCAAGACGAACCGGAA
AGTTACCGTGAAACAGCTCAAAGAAGACTATTTCAAAAAGATTGAATGTTTCGACTCTGTTGA
AATCAGCGGAGTGGAGGATCGCTTCAACGCATCCCTGGGAACGTATCACGATCTCCTGAAAAT
CATTAAAGACAAGGACTTCCTGGACAATGAGGAGAACGAGGACATTCTTGAGGACATTGTCCT
CACCCTTACGTTGTTTGAAGATAGGGAGATGATTGAAGAACGCTTGAAAACTTACGCTCATCTC
TTCGACGACAAAGTCATGAAACAGCTCAAGAGGCGCCGATATACAGGATGGGGGCGGCTGTC
AAGAAAACTGATCAATGGGATCCGAGACAAGCAGAGTGGAAAGACAATCCTGGATTTTCTTAA
GTCCGATGGATTTGCCAACCGGAACTTCATGCAGTTGATCCATGATGACTCTCTCACCTTTAAG

GAGGACATCCAGAAAGCACAAGTTTCTGGCCAGGGGGACAGTCTTCACGAGCACATCGCTAAT
CTTGCAGGTAGCCCAGCTATCAAAAAGGGAATACTGCAGACCGTTAAGGTCGTGGATGAACTC
GTCAAAGTAATGGGAAGGCATAAGCCCGAGAATATCGTTATCGAGATGGCCCGAGAGAACCA
AACTACCCAGAAGGGACAGAAGAACAGTAGGGAAAGGATGAAGAGGATTGAAGAGGGTATA
AAAGAACTGGGGTCCCAAATCCTTAAGGAACACCCAGTTGAAAACACCCAGCTTCAGAATGAG
AAGCTCTACCTGTACTACCTGCAGAACGGCAGGGACATGTACGTGGATCAGGAACTGGACATC
AATCGGCTCTCCGACTACGACGTGGATCATATCGTGCCCCAGTCTTTTCTCGCCGATGATTCTA
TTGATAATAAAGTGTTGACAAGATCCGATAAAAATAGAGGGAAGAGTGATAACGTCCCCTCAG
AAGAAGTTGTCAAGAAAATGAAAAATTATTGGCGGCAGCTGCTGAACGCCAAACTGATCACAC
AACGGAAGTTCGATAATCTGACTAAGGCTGAACGAGGTGGCCTGTCTGAGTTGGATAAAGCCG
GCTTCATCAAAAGGCAGCTTGTTGAGACACGCCAGATCACCAAGCACGTGGCCCAAATTCTCG
ATTCACGCATGAACACCAAGTACGATGAAAATGACAAACTGATTCGAGAGGTGAAAGTTATTA
CTCTGAAGTCTAAGCTGGTCTCAGATTTCAGAAAGGACTTTCAGTTTTATAAGGTGAGAGAGAT
CAACAATTACCACCATGCGCATGATGCCTACCTGAATGCAGTGGTAGGCACTGCACTTATCAA
AAAATATCCCGCCCTTGAATCTGAATTTGTTTACGGAGACTATAAAGTGTACGATGTTAGGAAA
ATGATCGCAAAGTCTGAGCAGGAAATAGGCAAGGCCACCGCTAAGTACTTCTTTTACAGCAAT
ATTATGAATTTTTTCAAGACCGAGATTACACTGGCCAATGGAGAGATTCGGAAGGCCCCACTT
ATCGAAACAAACGGAGAAACAGGAGAAATCGTGTGGGACAAGGGTAGGGATTTCGCGACAGT
CCGGAAGGTCCTGTCCATGCCGCAGGTGAACATCGTTAAAAAGACCGAAGTACAGACCGGAG
GCTTCTCCAAGGAAAGTATCAGACCGAAAAGGAACAGCGACAAGCTGATCGCACGCAAAAAA
GATTGGGACCCCAAGAAATACGGCGGATTCGTGTCTCCTACAGTCGCTTACAGTGTACTGGTTG
TGGCCAAAGTGGAGAAAGGGAAGTCTAAAAAACTCAAAAGCGTCAAGGAACTGCTGGGCATC
ACAATCATGGAGCGATCAAGCTTCGAAAAAAACCCCATCGACTTTCTCGAGGCGAAAGGATAT
AAAGAGGTCAAAAAAGACCTCATCATTAAGCTTCCCAAGTACTCTCTCTTTGAGCTTGAAAAC
GGCCGGAAACGAATGCTCGCTAGTGCGAGATTCCTGCAGAAAGGTAACGAGCTGGCACTGCCC
TCTAAATACGTTAATTTCTTGTATCTGGCCAGCCACTATGAAAAGCTCAAAGGGTCTCCCGAAG
ATAATGAGCAGAAGCAGCTGTTCGTGGAACAACACAAACACTACCTTGATGAGATCATCGAGC
AAATAAGCGAATTCTCCAAAAGAGTGATCCTCGCCGACGCTAACCTCGATAAGGTGCTTTCTG
CTTACAATAAGCACAGGGATAAGCCCATCAGGGAGCAGGCAGAAAACATTATCCACTTGTTTA
CTCTGACCAACTTGGGCGCGCCTAGAGCCTTCAAGTACTTCGACACCACCATAGACAGAAAGG
TGTACAGATCTACAAAGGAGGTCCTGGACGCCACACTGATTCATCAGTCAATTACGGGGCTCT
ATGAAACAAGAATCGACCTCTCTCAGCTCGGTGGAGACAGCAGGGCTGACCCCAAGAAGAAG
AGGAAGGTGTGAATCGATGGTACCTATGGGCACCAAAGAACCTGTAAACGTTATCTTTTTAAA
TTGAATGTGCACAAATAAAAGTTTGGAAAAGAAAAAAAAAAAAAAAAAAAAAAAAAAAAAAAA
AAAAAAAAAAAAAAAAAAAAAAAAAAAAAAAAAAAAAAAAAAAAAAAAAAAAAAAAAAAAAA
AAAAACCCTCGAGGTAGCATGC

>Amino acid sequence of eSpCas9-NG (L1111R/D1135V/G1218R/E1219F/A1322R/R1335V/T1337R + K848A/K1003A/R1060A)
MDYKDDDDKIMAPKKKRKVGIHGVPAADKKYSIGLDIGTNSVGWAVITDEYKVPSKKFKVLGNT
DRHSIKKNLIGALLFDSGETAEATRLKRTARRRYTRRKNRICYLQEIFSNEMAKVDDSFFHRLEESFL
VEEDKKHERHPIFGNIVDEVAYHEKYPTIYHLRKKLVDSTDKADLRLIYLALAHMIKFRGHFLIEGD

LNPDNSDVDKLFIQLVQTYNQLFEENPINASGVDAKAILSARLSKSRRLENLIAQLPGEKKNGLFGN
LIALSLGLTPNFKSNFDLAEDAKLQLSKDTYDDDLDNLLAQIGDQYADLFLAAKNLSDAILLSDILR
VNTEITKAPLSASMIKRYDEHHQDLTLLKALVRQQLPEKYKEIFFDQSKNGYAGYIDGGASQEEFY
KFIKPILEKMDGTEELLVKLNREDLLRKQRTFDNGSIPHQIHLGELHAILRRQEDFYPFLKDNREKIE
KILTFRIPYYVGPLARGNSRFAWMTRKSEETITPWNFEEVVDKGASAQSFIERMTNFDKNLPNEKVL
PKHSLLYEYFTVYNELTKVKYVTEGMRKPAFLSGEQKKAIVDLLFKTNRKVTVKQLKEDYFKKIEC
FDSVEISGVEDRFNASLGTYHDLLKIIKDKDFLDNEENEDILEDIVLTLTLFEDREMIEERLKTYAHLF
DDKVMKQLKRRRYTGWGRLSRKLINGIRDKQSGKTILDFLKSDGFANRNFMQLIHDDSLTFKEDIQ
KAQVSGQGDSLHEHIANLAGSPAIKKGILQTVKVVDELVKVMGRHKPENIVIEMARENQTTQKGQ
KNSRERMKRIEEGIKELGSQILKEHPVENTQLQNEKLYLYYLQNGRDMYVDQELDINRLSDYDVDH
IVPQSFLADDSIDNKVLTRSDKNRGKSDNVPSEEVVKKMKNYWRQLLNAKLITQRKFDNLTKAER
GGLSELDKAGFIKRQLVETRQITKHVAQILDSRMNTKYDENDKLIREVKVITLKSKLVSDFRKDFQF
YKVREINNYHHAHDAYLNAVVGTALIKKYPALESEFVYGDYKVYDVRKMIAKSEQEIGKATAKYF
FYSNIMNFFKTEITLANGEIRKAPLIETNGETGEIVWDKGRDFATVRKVLSMPQVNIVKKTEVQTGG
FSKESIRPKRNSDKLIARKKDWDPKKYGGFVSPTVAYSVLVVAKVEKGKSKKLKSVKELLGITIME
RSSFEKNPIDFLEAKGYKEVKKDLIIKLPKYSLFELENGRKRMLASARFLQKGNELALPSKYVNFLY
LASHYEKLKGSPEDNEQKQLFVEQHKHYLDEIIEQISEFSKRVILADANLDKVLSAYNKHRDKPIRE
QAENIIHLFTLTNLGAPRAFKYFDTTIDRKVYRSTKEVLDATLIHQSITGLYETRIDLSQLGGDSRAD
PKKKRKV

>gRNA1 for Tyrosinase (from T3 promoter to DraI site)
GAAATTAACCCTCACTAAAGGGTGGATGACCGTGAGTCCGTTTTAGTCCCTGAAAAGGGACTAAAATAAAG
AGTTTGCGGGACTCTGCGGGGTTACAATCCCCTAAAACCGCTTTTAAA

>gRNA2 for Tyrosinase (from T3 promoter to DraI site)
GAAATTAACCCTCACTAAAGGAATGCTGCCCACCATGGATGTTTTAGTCCCTGAAAAGGGACTAAAATAAA
GAGTTTGCGGGACTCTGCGGGGTTACAATCCCCTAAAACCGCTTTTAAA

>gRNA3 for Tyrosinase (from T3 promoter to DraI site)
GAAATTAACCCTCACTAAAGGTCATCCACCCCTTTGAAGGGTTTTAGTCCCTGAAAAGGGACTAAAATAAAG
AGTTTGCGGGACTCTGCGGGGTTACAATCCCCTAAAACCGCTTTTAAA

>gRNA4 for Tyrosinase (from T3 promoter to DraI site)
GAAATTAACCCTCACTAAAGGGGATGACATAGACTGAGCGTTTTAGTCCCTGAAAAGGGACTAAAATAAAG
AGTTTGCGGGACTCTGCGGGGTTACAATCCCCTAAAACCGCTTTTAAA

>gRNA5 for Tyrosinase (from T3 promoter to DraI site)
GAAATTAACCCTCACTAAAGGATGGGTGATGGGAGTCCCGTTTTAGTCCCTGAAAAGGGACTAAAATAAAG
AGTTTGCGGGACTCTGCGGGGTTACAATCCCCTAAAACCGCTTTTAAA

>gRNA6 for Tyrosinase (from T3 promoter to DraI site)
GAAATTAACCCTCACTAAAGGGAACTGAGGTCCAGATGGGTTTTAGTCCCTGAAAAGGGACTAAAATAAAG
AGTTTGCGGGACTCTGCGGGGTTACAATCCCCTAAAACCGCTTTTAAA

>gRNA7 for Tyrosinase (from T3 promoter to DraI site)
GAAATTAACCCTCACTAAAGGCTGTTTTGTATTGCCTTCGTTTTAGTCCCTGAAAAGGGACTAAAATAAAGA
GTTTGCGGGACTCTGCGGGGTTACAATCCCCTAAAACCGCTTTTAAA

>gRNA8 for Tyrosinase (from T3 promoter to DraI site)
GAAATTAACCCTCACTAAAGGCCAGGATATCCTTCTGTCCGTTTTAGTCCCTGAAAAGGGACTAAAATAAAG
AGTTTGCGGGACTCTGCGGGGTTACAATCCCCTAAAACCGCTTTTAAA

>gRNA9 for Tyrosinase (from T3 promoter to DraI site)
GAAATTAACCCTCACTAAAGGACTCCCATCACCCATCCATGTTTTAGTCCCTGAAAAGGGACTAAAATAAAG
AGTTTGCGGGACTCTGCGGGGTTACAATCCCCTAAAACCGCTTTTAAA

>gRNA for Nr6a1 (from T3 promoter to DraI site)
GAAATTAACCCTCACTAAAGGCACAGGTCACTCCTTCACGTTTTAGTCCCTGAAAAGGGACTAAAATAAAG
AGTTTGCGGGACTCTGCGGGGTTACAATCCCCTAAAACCGCTTTTAAA

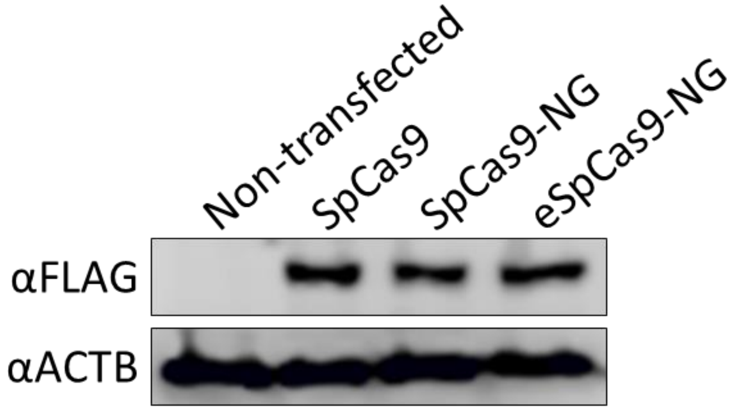

Supplementary Figure 2. Confirmation of Cas9 expression by immunoblotting.

A band with the expected molecular weight (about 162.8 kD) of the Cas9 protein was observed in HEK293 cells. β-actin (ACTB) is shown as a loading control.

(A)

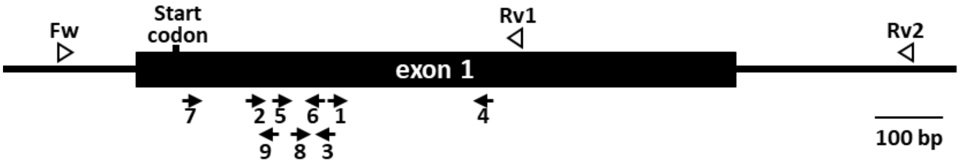

(B)

Cas9	gRNA	PAM	Target sequence	No. (%) of mutated/total blastocyst[1]				% of mutation efficiency by TIDE[2]
				1st experiment		2nd experiment		
SpCas9-NG	1	TGG	GGGTGGATGACCGTGAGTCCTGG	16/16	(100)	10/10	(100)	50.7
SpCas9-NG	2	GGG	GAATGCTGCCCACCATGGATGGG	11/11	(100)	12/13	(92.3)	42.8
SpCas9-NG	3	GGA	GGTCATCCACCCCTTTGAAGGGGA	12/12	(100)	21/21	(100)	58.2
SpCas9-NG	4	TGA	GGGGATGACATAGACTGAGCTGA	10/11	(90.9)	13/13	(100)	80.7
SpCas9-NG	5	TGC	GGATGGGTGATGGGAGTCCCTGC	10/11	(90.9)	12/14	(85.7)	36.1
SpCas9-NG	6	TGC	GGGAACTGAGGTCCAGATGGTGC	12/12	(100)	11/11	(100)	79.9
SpCas9-NG	7	TGT	GGCTGTTTTGTATTGCCTTCTGT	7/11	(63.6)	13/13	(100)	32.1
SpCas9-NG	8	AGT	GCCAGGATATCCTTCTGTCCAGT	16/16	(100)	11/11	(100)	95.4
SpCas9-NG	9	GGT	GGACTCCCATCACCCATCCATGGT	8/8	(100)	23/24	(95.8)	94.9
SpCas9	3	GGA	GGTCATCCACCCCTTTGAAGGGGA	4/16	(25.0)	1/12	(8.3)	2.2
SpCas9	9	GGT	GGACTCCCATCACCCATCCATGGT	1/8	(12.5)	0/8	(0)	2.6

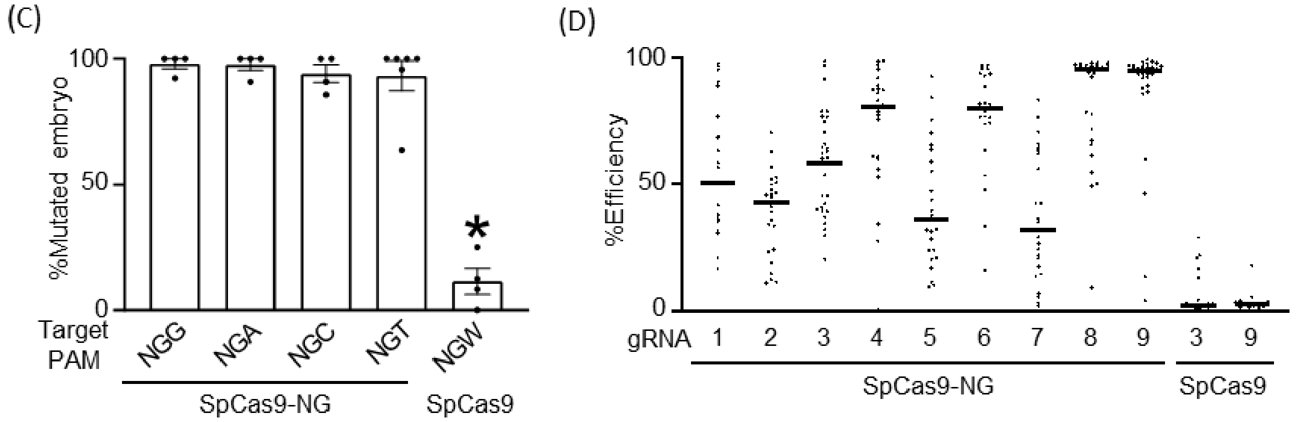

Supplementary Figure 3. Schematics of the target loci of each gRNA and summary of the obtained results on embryos.

(A) The gRNAs designed in the tyrosinase locus (Chr7: 87492138 to 87493665 in GRCm38.p4). Arrows indicate the gRNA-designed site and arrowheads indicate PCR primers. Rv2 was used only for the detection of mutation by gRNA-4. (B) Target sequences of each gRNA and the mutagenic efficiencies analyzed at the blastocyst stages. The gRNA-1 has been designed previously and usable for efficient knockout by wildtype SpCas9 [40]. [1]The blastocyst which showed the chromatogram data including the indels or multiple waveforms was counted as mutated. The representative data are shown in Supplementary Figure 4. [2]The median of the mutagenic efficiencies in each blastocyst measured by TIDE [34]. (C) The results from the same PAM (NGG, NGA, NGC or NGT with SpCas9-NG, and NGW with SpCas9 in (B)) were used to calculate. Statistical analyses of results were performed by ANOVA with a Bonferroni/Dunn post hoc test, and the mutagenic rate by SpCas9 is significantly lower than the rates by SpCas9-NG in each PAM (*$P<0.05$). (D) Mutagenic efficiencies measured by TIDE. Dots indicate the mutation efficiencies measured by TIDE using Sanger sequence chromatogram data from each blastocyst, and black bars indicate the median of the efficiencies.

[SpCas9-NG + gRNA-1]

GGGTGGATGACCGTGAGTCCTGG

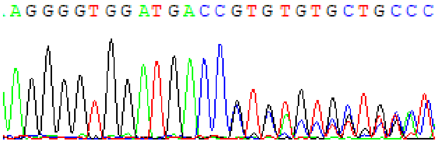

[SpCas9-NG + gRNA-2]

GAATGCTGCCCACCATGGATGGG

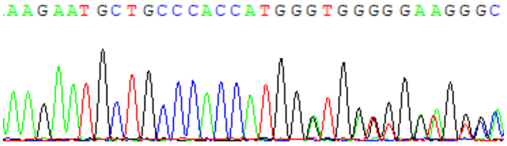

[SpCas9-NG + gRNA-3]

GGTCATCCACCCCTTTGAAGGGGA*

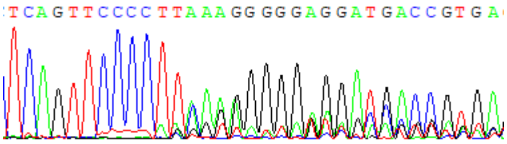

[SpCas9-NG + gRNA-4]

GGGGATGACATAGACTGAGCTGA*

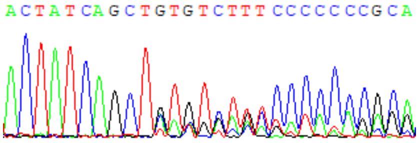

[SpCas9-NG + gRNA-5]

GGATGGGTGATGGGAGTCCCTGC

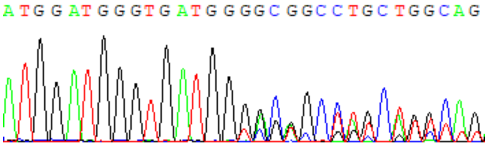

[SpCas9-NG + gRNA-6]

GGGAACTGAGGTCCAGATGGTGC*

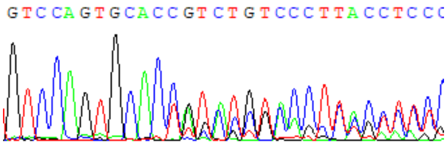

[SpCas9-NG + gRNA-7]

GGCTGTTTTGTATTGCCTTCTGT

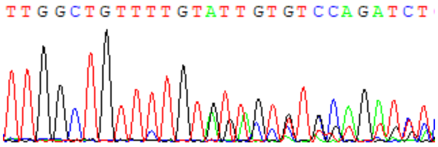

[SpCas9-NG + gRNA-8]

GCCAGGATATCCTTCTGTCCAGT

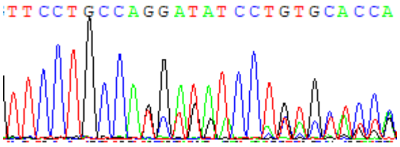

[SpCas9-NG + gRNA-9]

GGACTCCCATCACCCATCCATGGT

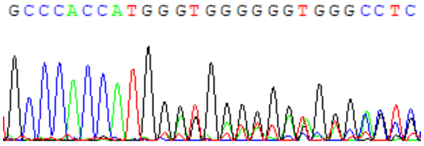

[SpCas9 + gRNA-3]

GGTCATCCACCCCTTTGAAGGGGA*

Mutated

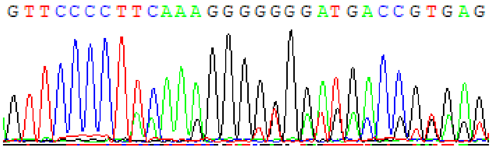

Unmodified

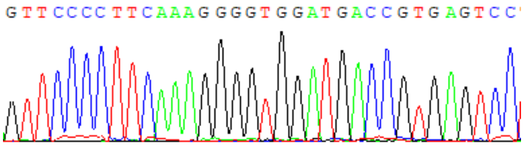

[SpCas9 + gRNA-9]

GGACTCCCATCACCCATCCATGGT

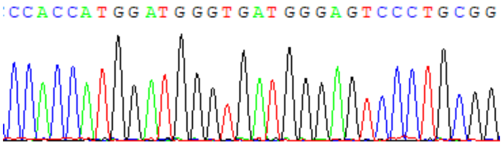

Supplementary Figure 4. Direct sequencing analysis of tyrosinase-targeted blastocysts. Representative image of the waveform data obtained by PCR-directed Sanger sequencing. The upper nucleotides indicate the wildtype sequence of the target site and underlining shows the PAM. *The sequence is a reverse-complement strand.

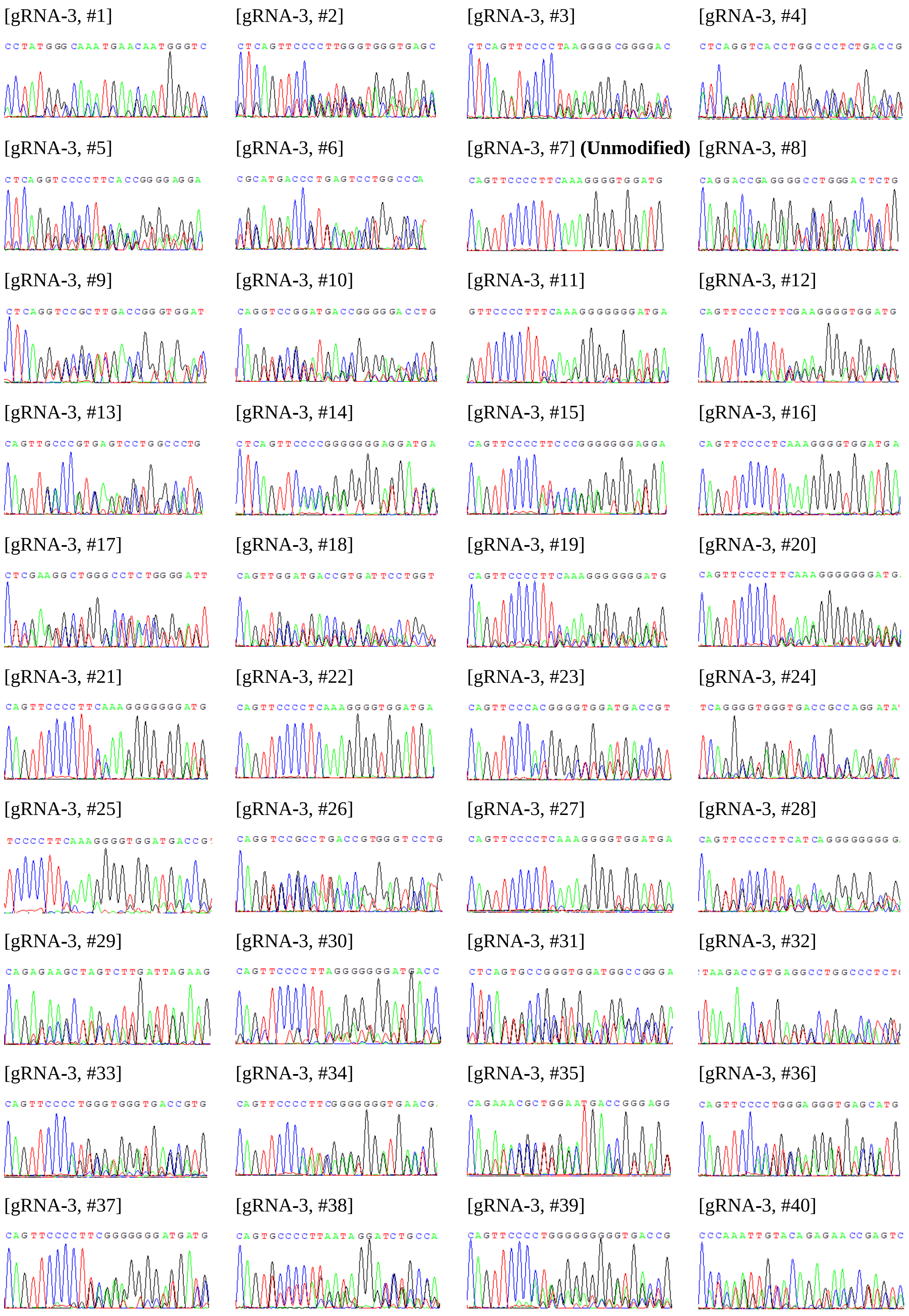

Supplementary Figure 5. Direct sequencing analysis of tyrosinase-targeted pups using gRNA-3.

[gRNA-9, #1]

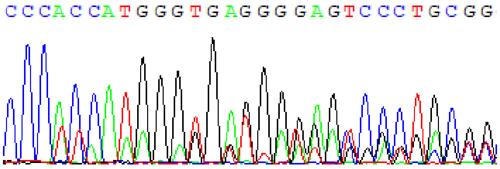

[gRNA-9, #2]

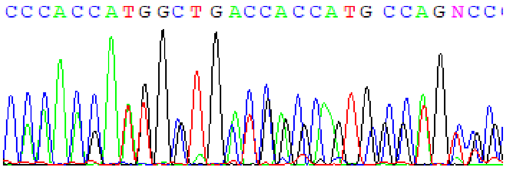

[gRNA-9, #3]

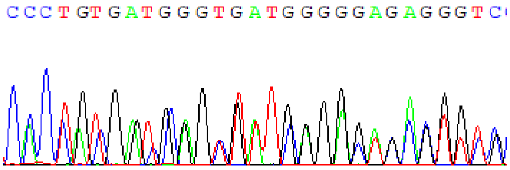

[gRNA-9, #4]

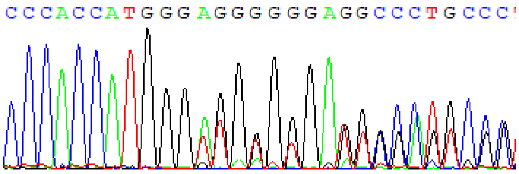

[gRNA-9, #5]

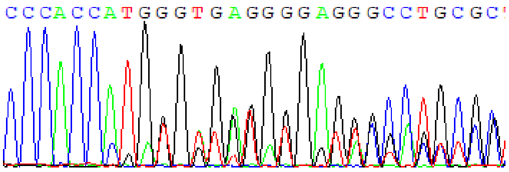

Supplementary Figure 6. Direct sequencing analysis of tyrosinase-targeted pups using gRNA-9.

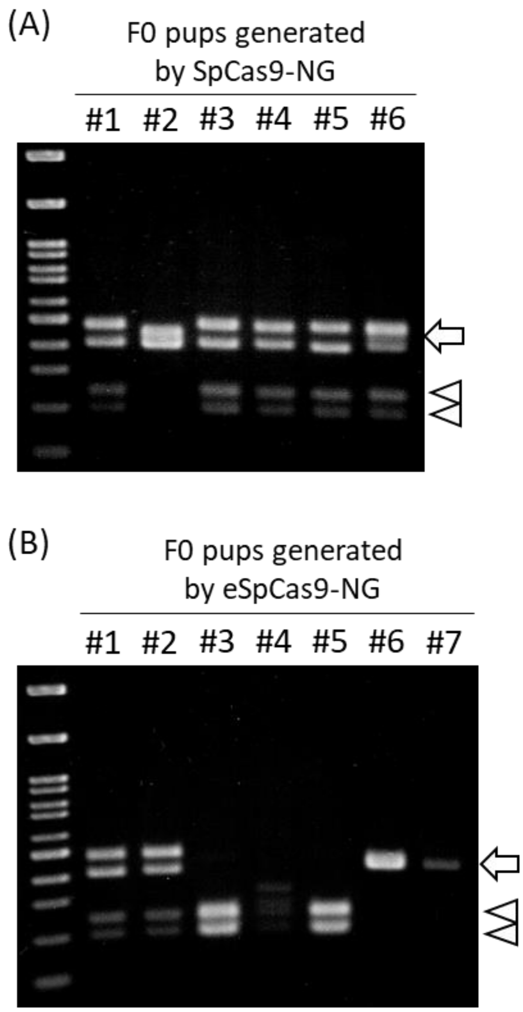

Supplementary Figure 7. Nr6a1-Flag knock-in F0 pups detected by PCR-RFLP.

The knock-in allele was detected by BamHI-restriction fragment length polymorphism using the genomic PCR amplicons of Nr6A1-modified mice generated by SpCas9-NG (A) and eSpCas9-NG (B). An arrow indicates undigested fragments and arrowheads indicate digested fragments.

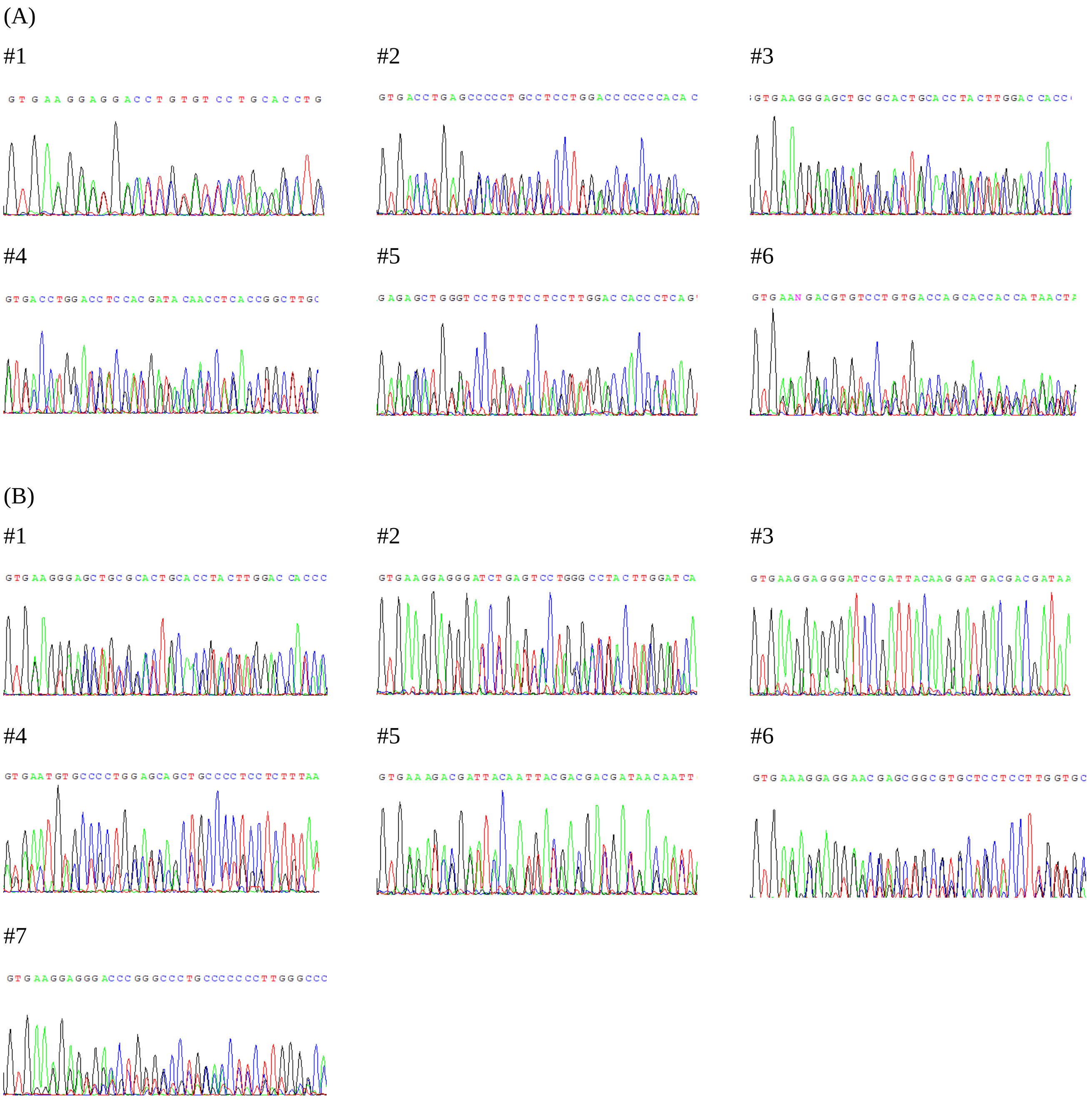

Supplementary Figure 8. Direct sequencing analysis of Nr6a1-targeted pups.

Waveform data obtained by PCR-directed sequencing of Nr6a1-modified mice generated by SpCas9-NG (A) and eSpCas9-NG (B).

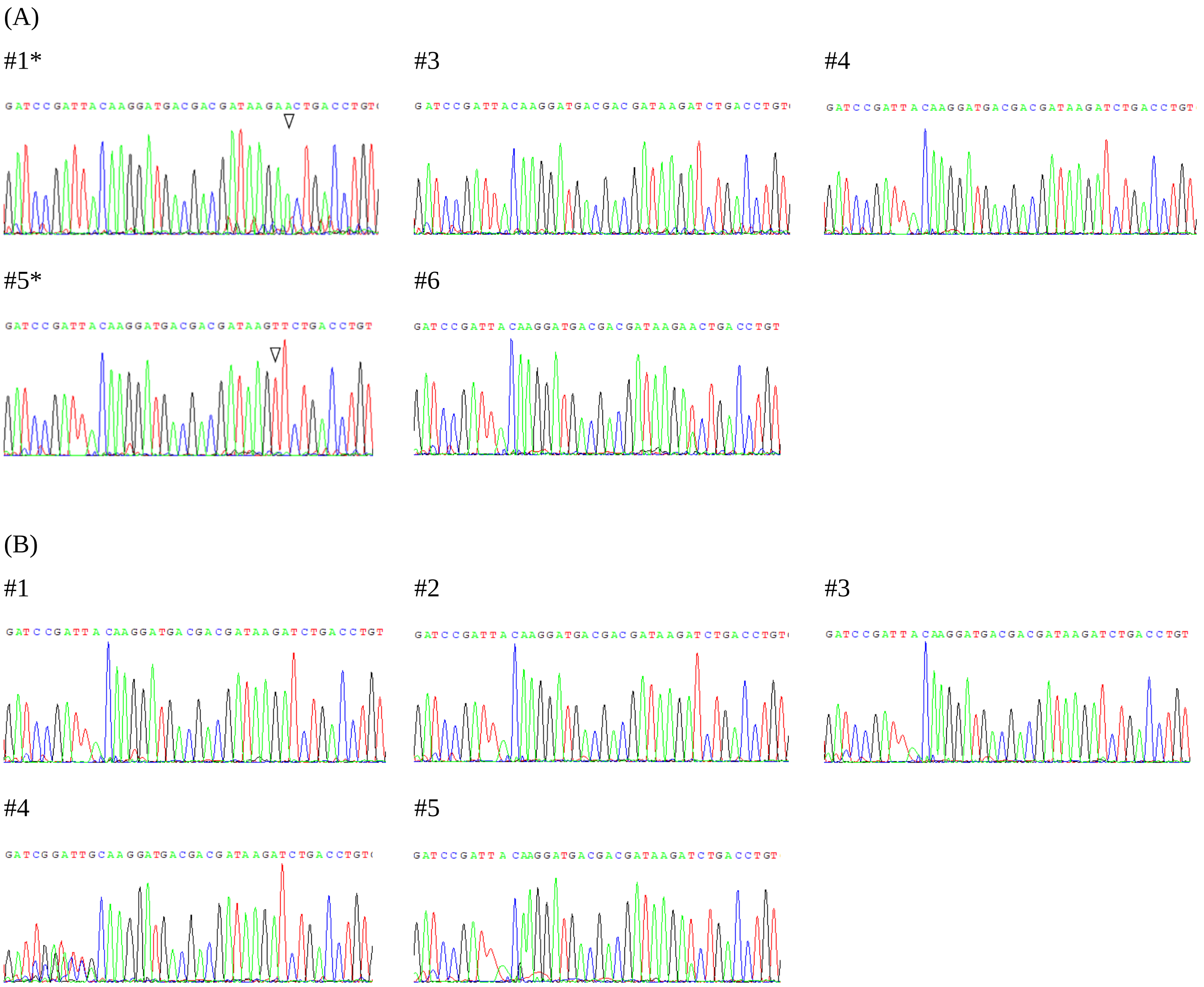

Supplementary Figure 9. Flag-tag knock-in detected by sequencing of digested PCR fragments. Waveform data of the Flag-tag knock-in allele in Nr6a1-modified mice generated by SpCas9-NG (A) and eSpCas9-NG (B). The BamHI-digested fragments (shown in Supplementary Figure 7) were extracted and sequenced by using the Nr6a1 reverse primer shown in Supplementary Table 1. *An arrowhead indicates a nucleotide-substituted site occurring between the FLAG-tag coding sequence and stop codon.

#2

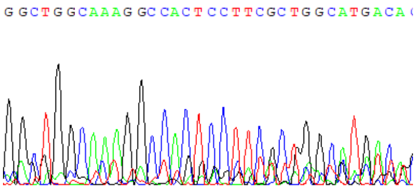

#5

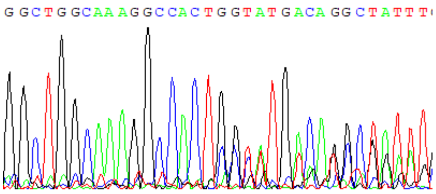

#6

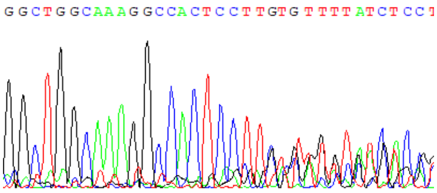

Supplementary Figure 10. Off-target mutations of Nr6a1-Flag F0 pups.

Waveform mutation sequence data of off-target site 2 in Nr6a1-modified pups generated by using SpCas9-NG. Off-target locus information is shown in Supplementary Table 2.

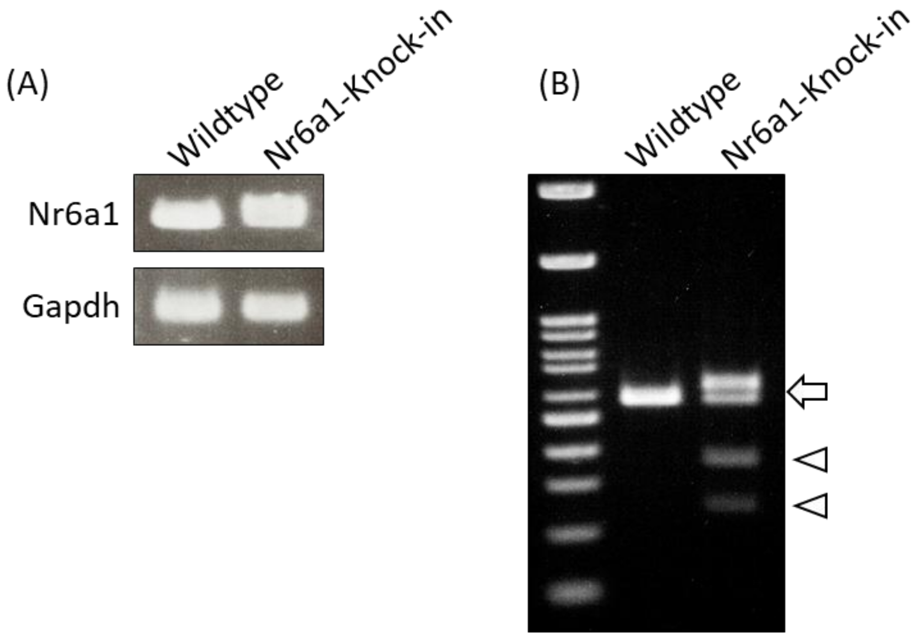

Supplementary Figure 11. RT-PCR of Nr6a1-Flag knock-in mice.

(A) RT-PCR using testicular cells derived from a wildtype and a F0 Nr6a1-Flag knock-in pup.

(B) The knock-in allele-derived transcription was detected by BamHI-restriction fragment length polymorphism using the Nr6a1 RT-PCR amplicons (A). An arrow indicates undigested fragments and arrowheads indicate digested fragments.

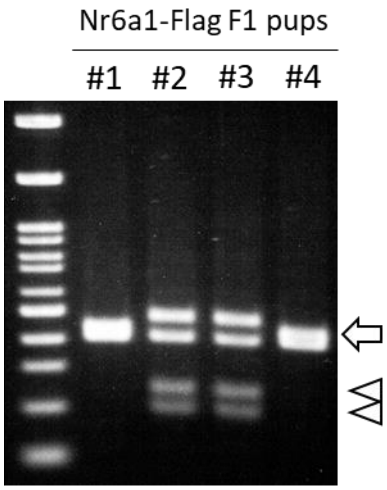

Supplementary Figure 12. PCR-RFLP of Nr-6a1-Flag F1 pups.

The knock-in allele was detected by BamHI-restriction fragment length polymorphism using the genomic PCR amplicons of Nr6A1-Flag F1pups. An arrow indicates undigested fragments and arrowheads indicate digested fragments.

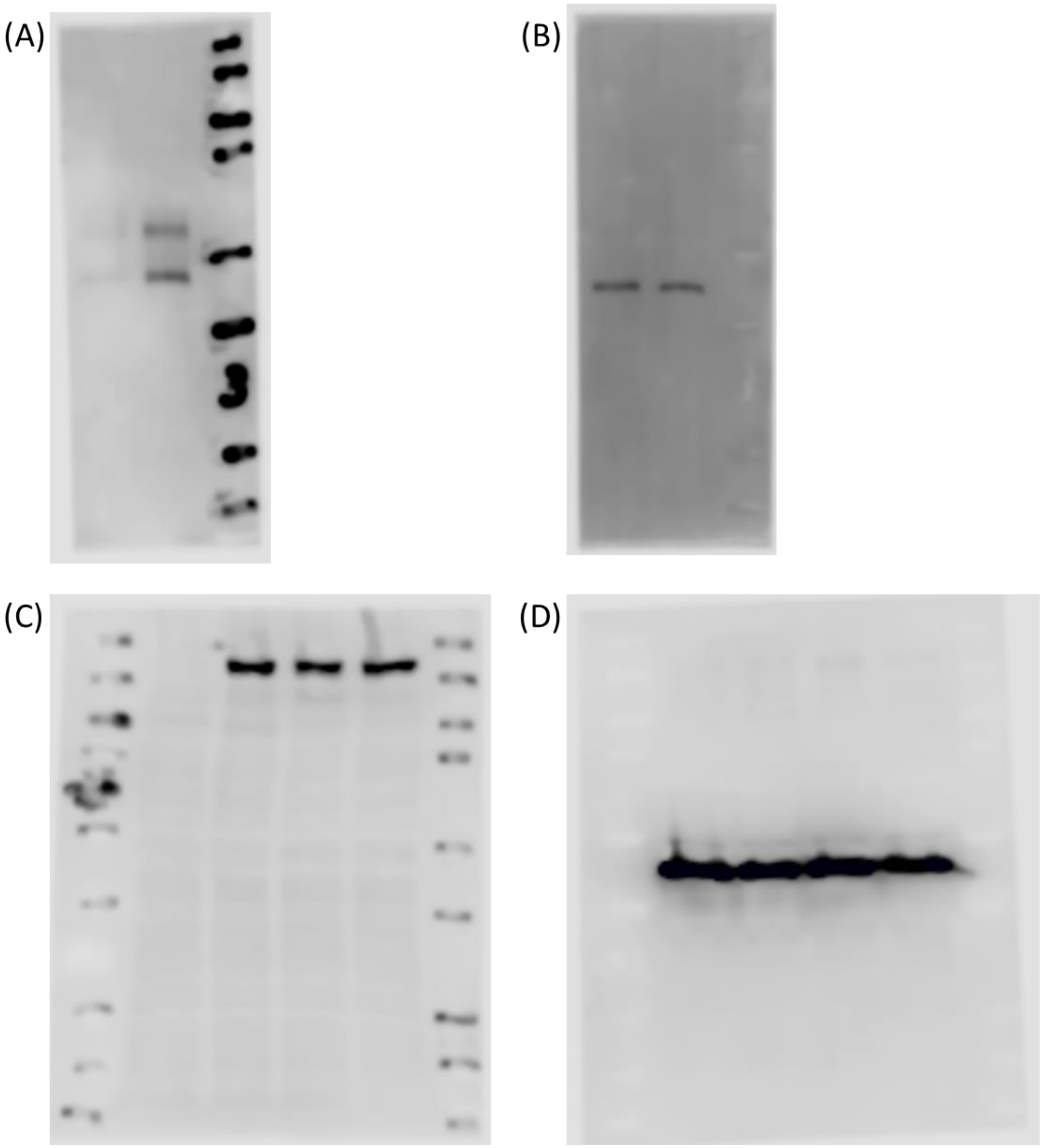

Supplementary Figure 13. Full-length Western blot images.

(A) Full-length blot image for anti-FLAG in Figure 2D. (B) Full-length blot image for anti-ACTB in Figure 2D. (C) Full-length blot image for anti-FLAG in Supplementary Figure 2. (D) Full-length blot image for anti-ACTB in Supplementary Figure 2.

Supplementary Table 1. Primer sets for genomic PCR.

Target locus			Sequences
Tyrosinase target	Forward	5'-	TTATGCATTGAAGCAGTTCACC
	Reverse1	5'-	TTTGACAGTGGTGGAAACTGTCC
	Reverse2	5'-	AGCAATGTTACTTCGCAGCAGAGC
Nr6a1 genomic PCR	Forward	5'-	TACACTTGCCCTCATCCTGGAC
	Reverse	5'-	TGCATCATGTTGAAGGCCATAC
Nr6a1 off-target 1	Forward	5'-	CTGCACATGTCATCAAAATCAACCC
	Reverse	5'-	TGAAGGTGCAGACTCAAAGATCC
Nr6a1 off-target 2	Forward	5'-	CTGCTGCAGAAGGGTCAAAGTTC
	Reverse	5'-	ATTCAATCCCTGAAGCCTGGATG
Nr6a1 ssODN		5'-	GCCCCTCCTCTTTAAGGTGGTGCTGCACTCCTGC AAGACAAGTACGGTGAAGGAGGGATCCGATTAC AAGGATGACGACGATAAGATCTGACCTGTGCCCT GCACCTCCTTGGGCCACCCACAGTGCCTTGGGTA GGCAGCACAGGCTCC
Nr6a1 RT-PCR	Forward	5'-	GGGATGGAGGTGATTGAACGAC
	Reverse	5'-	TGCATCATGTTGAAGGCCATAC
Gapdh RT-PCR	Forward	5'-	GTGCTGAGTATGTCGTGGAGTC
	Reverse	5'-	CATACTTGGCAGGTTTCTCCAG

Supplementary Table 2. List of potential off-target loci for Nr6a1 on-target sequence.

Off-target loci in the chromosome 2, which is same as the Nr6a1, was marked by yellow.

off-target Sequence	mismatch	chr	start	end	locusDesc
**GGAACAGGTCAGTCCTTCACAGT**	**2**	**chr4**	**66563148**	**66563170**	**intergenic:Gm25480-Gm11220**
**GGCAAAGGCCACTCCTTCACTGG**	**2**	**chr17**	**69304961**	**69304983**	**intergenic:Epb4.1l3-Zbtb14**
TGCACAGAGCACTCCTTCACTGA	3	chr1	97748208	97748230	intron:Ppip5k2
GACACAGGTCCTTCCTTCACAGA	3	chr1	78549	78571	intergenic:A530040E14RIK-AC125149.4
GACACAGGTCCTTCCTTCACAGA	3	chr1	182729	182751	intergenic:Csprs-Gap
GACACAGGTCCTTCCTTCACAGA	3	chr1	24289	24311	intergenic:Gap-AC168977.2
GACACAGGTCCTTCCTTCACAGA	3	chr1	85031757	85031779	intron:Gm7582
GACACAGGTCCTTCCTTCACAGA	3	chr1	521	543	intergenic:Gap-AC133103.4
GACACAGGTCCTTCCTTCACAGA	3	chr1	85306891	85306913	intergenic:Gm16026-Gm2619
GACACAGGTCCTTCCTTCACAGA	3	chr1	85209711	85209733	intron:Gm7609
GACACAGGTCCTTCCTTCACAGA	3	chr1	85546833	85546855	intron:Gm7592
GACACAGGTCCTTCCTTCACAGA	3	chr1	218974	218996	intergenic:AC133103.1-AC133103.3
GACACAGGTCCTTCCTTCACAGA	3	chr1	525	547	intergenic:Gap-AC132444.1
GACACAGGTCCTTCCTTCACAGA	3	chr1	84960707	84960729	intergenic:Slc16a14-Gm15433
GACACAGGTACCTCCTTCACAGC	3	chr1	15712071	15712093	exon:Kcnb2
GGCAGATGTCACGCCTTCACTGT	3	chr1	89677210	89677232	intergenic:Agap1-Gm26037
GCCACAGGTCACTCCTCCAGAGG	3	chr2	19406961	19406983	intergenic:Msrb2-Gm13344
CTCACAGGTCACTCCATCACTGA	3	chr2	78534202	78534224	intergenic:Gm14460-Gm23149
TGAACAGGTCACTCCTACACAGC	3	chr3	124804883	124804905	intergenic:1700003H04Rik-Gm23673
GGCACAGCTCAGTCCATCACAGG	3	chr4	108971192	108971214	intron:8030443G20Rik
TACACAGGTCACTCCTGCACTGT	3	chr4	56306063	56306085	intergenic:2310081O03Rik-Gm12518
GGCATAGGTCACTCCATCTCTGA	3	chr5	35016965	35016987	intron:Rgs12
GGCAGCGCTCACTCCTTCACAGT	3	chr5	108002217	108002239	intergenic:Fam69a-Gm9850
GGCATAGTTCACTGCTTCACTGT	3	chr5	61642302	61642324	intergenic:Gm23708-G6pd2
GGCACAGGCCACTCTCTCACTGG	3	chr6	113548779	113548801	intron:Fancd2
TGCACTGGTCACTCCTTTACAGT	3	chr6	119648702	119648724	intron:Erc1
GGTACAGGTCACTCCTGCATTGG	3	chr7	100650322	100650344	intron:Plekhb1
GACACAGGTTACACCTTCACTGC	3	chr7	144518034	144518056	intron:Ppfia1
TGCACAGATCACTCCTTCGCTGC	3	chr7	131679843	131679865	intergenic:Gm16477-Gpr26
GGCACAGGGCATCCCTTCACAGT	3	chr7	122955369	122955391	intergenic:Cacng3-Rbbp6
GCCACATGTCACTCCTTCAGAGC	3	chr7	97714181	97714203	intron:Clns1a
GGCACAGGTCCCTCCTTGAGGGT	3	chr7	117572237	117572259	intron:Xylt1
GGCACAGGGCACTCGTTCAATGG	3	chr8	29029845	29029867	intron:Unc5d
AGCACAGGTCACTCACTCACTGC	3	chr9	115986721	115986743	intergenic:Gadl1-Gm16142
GGCACAGGGCTCTCCTTAACTGG	3	chr10	114577615	114577637	intron:Trhde
GGCACCTGGCACTCCTTCACAGA	3	chr10	61014876	61014898	intergenic:Mir466j-Gm20611
AGCACATGTCTCTCCTTCACAGG	3	chr11	75854478	75854500	exon:Rph3al
GACACAGGTCAAACCTTCACAGA	3	chr11	34114231	34114253	intergenic:4930469K13Rik-Gm12121
GGCACAGGTCACAACCTCACAGC	3	chr11	34411632	34411654	intron:Fam196b

GGCACAGCTCACTGTTTCACTGC	3	chr12	36184193	36184215	intron:Ankmy2
GGCACAGGGCCCTCCTTCATAGC	3	chr13	37302837	37302859	intergenic:F13a1-Ly86
GGCACAGGCCACTGCTCCACAGT	3	chr13	30405414	30405436	intergenic:Gm11369-Uqcrfs1
GGCAAAGGTCACCCATTCACTGA	3	chr14	27344777	27344799	intron:Arhgef3
GACACAGGACAATCCTTCACAGG	3	chr15	24503460	24503482	intergenic:Gm23980-Gm22810
GGTACAGGTCACTCCTGCATGGC	3	chr15	99678994	99679016	intron:Asic1
AGCACGGGTCACTACTTCACAGC	3	chr16	44968235	44968257	intron:Cd200r3
CGTCCAGGTCACTCCTTCACAGA	3	chr17	31737936	31737958	intergenic:Cryaa-Sik1
GGCTCAGGCCACTCCTACACTGC	3	chr18	37845140	37845162	exon:Diap1
AGCACAGGACACTCTTTCACAGC	3	chr18	72108629	72108651	intron:Dcc
GACACCGGTCACTGCTTCACTGT	3	chr18	74715859	74715881	intron:Myo5b
GGCACAGATCACTCCTTTCCTGG	3	chr19	16223312	16223334	intergenic:Gnaq-Gm10819
GGCACAGGGCACTCCCTCCCTGT	3	chr19	59264989	59265011	intron:Slc18a2
GGCAAAGGTCACTTCTACACTGG	3	chrX	121011629	121011651	intergenic:Gm4993-Gm22590
GGCACAGGCTACTCCTTCAGAGG	3	chrX	113743062	113743084	intron:Dach2
GGCTCAGTTCACACCATCACTGG	4	chr1	21251886	21251908	intron:Gsta3
AGCAGTGGTCACTCCTTGACAGG	4	chr1	185805733	185805755	intergenic:Slc30a10-Lyplal1
GGCACAGGACACCCCTGCTCAGG	4	chr1	39640553	39640575	intron:Creg2
GGCTCAGATGACTCCTGCACAGG	4	chr1	64531388	64531410	intergenic:Gm25748-Creb1
GGCACTGTCCACTCCTTCAGGGG	4	chr1	155018387	155018409	intergenic:Cacna1e-Gm9530
GGCTCAGGTCAATTCTCCACAGA	4	chr1	20652546	20652568	intergenic:Pkhd1-Mir206
AGCACAGCTCAGTTCTTCACAGA	4	chr1	94499346	94499368	intergenic:Pdcd1-Gm23389
GGCATAGCTGACTCTTTCACCGG	4	chr1	93116202	93116224	intergenic:Kif1a-Agxt
GGCACGGGAGACTCCTTCTCAGA	4	chr1	156222309	156222331	intergenic:Fam163a-Tdrd5
GGCACCGGGAACTCCTTAACTGA	4	chr1	33449003	33449025	intergenic:Gm23453-Prim2
GGCTCAGCTCCCCCCTTCACTGA	4	chr1	133382075	133382097	intergenic:Etnk2-Sox13
GGCTCAGGCCACTCCATGACTGA	4	chr1	165795898	165795920	intergenic:Gm16565/Cd247-Gm23402
GCCTTAGGTCACTCCCTCACTGC	4	chr1	174475992	174476014	intergenic:Olfr220-Fmn2
GGCACTGGGCACTCATTAACTGA	4	chr1	131068532	131068554	intergenic:Mapkapk2-Gm25549
GGTAAAAGTCACTCCTTAACAGT	4	chr1	157117461	157117483	intergenic:Tex35-Rasal2
AGCACAGGTTACTCTTTCAGAGG	4	chr1	156547863	156547885	intergenic:Gm10031-Abl2
GCCACTGGTCACTCCGCCACAGC	4	chr1	92616726	92616748	intergenic:Olfr1410-Olfr12
GACACACTTCAGTCCTTCACTGT	4	chr1	130821054	130821076	intron:Gm15848
AGCACTGGTTACTCCTTCAGAGA	4	chr1	190095218	190095240	intergenic:Smyd2-Prox1
GGCACTGGTCACTCTTCCAGGGG	4	chr1	86346738	86346760	exon:Ncl
GGCTCAGGTTACACCTTCCCAGC	4	chr1	152845523	152845545	exon:Smg7
GGCCCAGGTGACTTCTCCACAGC	4	chr1	86055550	86055572	intergenic:2810459M11Rik-Psmd1
GGCACGGTTCTCTCCTTTACTGT	4	chr1	71501748	71501770	intergenic:Abca12-Atic
ATCACAGGTCGCTCATTCACAGT	4	chr1	189089920	189089942	intergenic:Kctd3-Kcnk2
GGCACAGATCACTTCTGGACAGA	4	chr1	8177818	8177840	intergenic:Gm26901-Sntg1
GGCACAGGGTGCTCTTTCACAGT	4	chr1	41905115	41905137	intergenic:Gm5973-Gm9915
GGCACTGCGCACTCGTTCACCGC	4	chr1	95498598	95498620	exon:Gm15427

GGCTCAGGACACTCCTTCTGGGT	4	chr1	193355201	193355223	intron:Camk1g
GGCAGAGGTCACACCTGCCCCGC	4	chr1	133310082	133310104	intergenic:Golt1a/Kiss1/GOLT1A-GOLT1A/Golt1a
GTCACAGGTCACTCTTTCTATGT	4	chr1	9794049	9794071	intergenic:1700034P13Rik-Sgk3
GCCCCAGGACACTCGTTCACAGT	4	chr1	86496241	86496263	intergenic:1700019O17Rik-Ptma
GGCCCAGGTCTGTCCTTCAGTGC	4	chr1	121332239	121332261	intron:Insig2
CTCACAGGTCACTGCTGCACGGG	4	chr1	65169684	65169706	intron:Idh1
GACACATGTCACTGATTCACTGT	4	chr1	154615061	154615083	intron:Cacna1e
GGTGCAGGCCACTGCTTCACTGC	4	chr1	179140866	179140888	intron:Smyd3
GGCACATGTCACTGCTGCAAGGA	4	chr1	136744415	136744437	intergenic:Gm16880-Gm22132
AGCAGAGATCACTGCTTCACTGT	4	chr1	159584008	159584030	intergenic:Rfwd2-Gm10530
TGCATTGGTCACTCCTACACAGG	4	chr2	105302638	105302660	intron:0610012H03Rik
GCCACAGGCCACACCTTCAAAGG	4	chr2	172016396	172016418	intergenic:Gm14641-Cbln4
CGCACAGCTCACTTCTTCAAAGG	4	chr2	37712908	37712930	intergenic:Strbp-Crb2
GGCATGGCTCACTCCTTCTCAGG	4	chr2	159766283	159766305	intergenic:Gm14219-Gm11445
TGCACAGCTCACTCACTCACAGG	4	chr2	74637208	74637230	intergenic:Gm13672-Evx2
GGCGCAGGGCTCTTCTTCACTGG	4	chr2	49751183	49751205	intron:Kif5c
GGCACAGGTTCCTTCTTGACTGG	4	chr2	76825542	76825564	exon:Ttn
ACCACAGGACACTCCTACACAGA	4	chr2	43053495	43053517	intergenic:Gm24350-Gm13464
GGAGGAGGTCACTCCTTCAGTGG	4	chr2	115440549	115440571	intergenic:Gm13976-3110099E03Rik
CTCTCAGGTCACTCCATCACTGA	4	chr2	29460713	29460735	intergenic:Gm24976-Med27
GTCACAGGTCACACCAACACAGA	4	chr2	159086944	159086966	intergenic:Dhx35-Gm25090
AGCACACGTGACTTCTTCACAGA	4	chr2	30378612	30378634	exon:Fam73b
AGCACAGGAAACTCATTCACTGA	4	chr2	166932129	166932151	exon:Cse1l
GGCACAGGGCAGCTCTTCACAGA	4	chr2	146596004	146596026	intergenic:4933406D12Rik-Gm14111
GGGACAGGTCATTCCTCCTCTGA	4	chr2	93539791	93539813	intergenic:Gm10804-Gm10803
GGCACTGTTCCCTCCTTCTCTGA	4	chr2	117660855	117660877	intergenic:Gm13982-Gm13985
GGCTGAGGTCACTCCTTTGCAGA	4	chr2	168011274	168011296	exon:Gm14236
GGCACAGGACACTTCATCTCAGT	4	chr2	132163142	132163164	intron:Gm14051
GGCATGTGTCACTCATTCACTGA	4	chr2	107132928	107132950	intergenic:Gm13903-Kcna4
TTCAAAGGTCACTCCCTCACAGT	4	chr2	143772074	143772096	intergenic:Gm12360/Pcsk2-Pcsk2
GGCCCAGATCAGTCCTTCATTGA	4	chr2	98507934	98507956	intergenic:Gm13804-Gm13806
GGCTCAGCTCTCTCCTTCGCAGA	4	chr2	162698448	162698470	intergenic:Gm14246-Gm22936
AGCTCAGGTTACTCTTTCACTGA	4	chr2	79882874	79882896	intron:Pde1a
GACAGAGCTGACTCCTTCACGGT	4	chr2	167205650	167205672	intron:Ptgis
AACACAGGTCACTTGTTCACAGA	4	chr2	70412600	70412622	intron:Myo3b
GTCAGAGGTATCTCCTTCACTGT	4	chr2	115673478	115673500	intron:BC052040
GGGACAGGCCACTCCTACATAGC	4	chr2	156848134	156848156	intron:Tgif2
GGCTAAGGTGAGTCCTTCACTGC	4	chr2	122587520	122587542	intergenic:Gm14085-Gatm
AGGACAGGTCACTCCCTCCCTGC	4	chr2	158333119	158333141	intergenic:Lbp-9430008C03Rik
TGCACAGGTAACTCCTTCCTTGC	4	chr2	166667586	166667608	intergenic:Gm23152-Gm25265
GGGACAGGTGGCTACTTCACAGC	4	chr2	32167735	32167757	intergenic:Prrc2b-AL808027.1
GGCATAGGTCCCTCTTCCACAGA	4	chr2	82993923	82993945	exon:Fsip2

GGCACAGTTCACTCTTTCTGGGA	4	chr2	70162866	70162888	intron:Myo3b
GGCACAGGTCTTGCCTGCACTGT	4	chr2	37509540	37509562	intergenic:Rabgap1-Gpr21
GGCACAGTACACTGCCTCACAGT	4	chr2	47582941	47582963	intergenic:Gm25959-Gm13468
GGCACTGGTGACTGCTCCACAGT	4	chr2	30962318	30962340	intron:Tor1a
GGCACAGGTCACAGCTTAGCTGG	4	chr2	26470417	26470439	exon:Notch1
GCCTCAGCTCACTGCTTCACAGT	4	chr2	93167028	93167050	intergenic:Gm13802-Trp53i11
GGCACATGTCATTGCTTCTCAGA	4	chr2	155737747	155737769	intergenic:Gm17581-Procr
GACACAGGTCACTGCTGCTCAGC	4	chr2	60724779	60724801	intergenic:Itgb6-Rbms1
GGCACACATCATTCCTTAACTGG	4	chr3	119172376	119172398	intron:Dpyd
GTCAGAGCTCAATCCTTCACAGG	4	chr3	79114254	79114276	intron:Rapgef2
GTCACAGGGCACTCACTCACTGG	4	chr3	82380803	82380825	intron:Map9
GTCACAGGTCACCCGATCACTGG	4	chr3	31505579	31505601	intergenic:Gm15496-Kcnmb2
GACACAGCTCCCTTCTTCACGGA	4	chr3	50372459	50372481	intron:Slc7a11
GGCACAAGTCACTATGTCACAGG	4	chr3	97809105	97809127	intron:Pde4dip
GAGAAAGGTGACTCCTTCACAGA	4	chr3	97730934	97730956	intron:Pde4dip
AGCATATGTAACTCCTTCACAGC	4	chr3	68802880	68802902	intergenic:Gm10040-Gm17641/1110032F04Rik
GGCTCAAGACAGTCCTTCACAGC	4	chr3	68430444	68430466	intergenic:Gm10292-Schip1
GACAATGGTCACTCCTTCCCAGA	4	chr3	7358712	7358734	intergenic:Gm22074-Pkia
GGCAGATGTCCCTCCTTCAATGA	4	chr3	102648278	102648300	intergenic:Ngf-2410057H14Rik
GGCACAAGTCACACCCACACAGT	4	chr3	9236320	9236342	intergenic:Tpd52-Zbtb10
GCCAAAGGCCACTCCTTGACTGA	4	chr3	156170807	156170829	intergenic:Gm23038-4930570G19Rik
AGCACAGGCCACTCTTTCAGAGG	4	chr3	133277078	133277100	intergenic:Arhgef38-Ppa2
CTCACAGGTCCCTCCTTTACTGC	4	chr3	35413378	35413400	intergenic:Gm25442-Gm25696
GATGCAGGTCCCTCCTTCACAGT	4	chr3	89668146	89668168	intergenic:Kcnn3-Adar
GGCATAACTCACTCATTCACTGC	4	chr3	125759106	125759128	intergenic:Ndst4-Ugt8a
AGCACAGGACAGTCTTTCACAGA	4	chr3	10242105	10242127	intergenic:Fabp4-Fabp12
AGCACAGGCCACTCGTTCTCAGA	4	chr3	143877483	143877505	intergenic:Gm24728-Lmo4
GTCCCAGGTCACTCCTACTCTGC	4	chr3	42498125	42498147	intergenic:D3Ertd751e-Gm25714
GGCAGAGGACACGCCTTCTCTGT	4	chr3	58832645	58832667	intergenic:4930593A02Rik-Clrn1
GGCACAGGTCAGTACCTAACAGC	4	chr3	120312232	120312254	intergenic:Gm26137-Gm23733
GGCTTAGGTCACTCCTACCCAGC	4	chr3	83800162	83800184	intergenic:Gm26771-Tlr2
GGCACAGGGCAGTCCTTGGCGGA	4	chr3	121850481	121850503	intergenic:Abcd3-Arhgap29
CACACAGATCACTCTTTCACAGC	4	chr3	155194874	155194896	intergenic:Lrriq3-Gm23038
CACACAGGTCCCTCCTTCCCTGT	4	chr3	110914435	110914457	intergenic:Gm26076-Gm25519
GACACAGCTCACTTCTTCAGTGC	4	chr3	149520461	149520483	intergenic:Gm26468-Rpsa-ps10
GTCATAGGTCACCCTTTCACTGC	4	chr3	42094946	42094968	intergenic:D3Ertd751e-Gm25714
GGCTTAGCTCACTCCTGCACTGT	4	chr3	60888389	60888411	intergenic:Gm8325-P2ry1
GGCACAAGTCCCTCCTTGTCAGT	4	chr3	10583165	10583187	intergenic:Snx16-Gm22795
AGCTCAGATCACTCGTTCACTGT	4	chr3	5138921	5138943	intergenic:Gm17308-2700069I18Rik
GCCACAGGTCACTCTTTTCCCGC	4	chr3	123476455	123476477	intron:Prss12
GACAGAGGTCACTCCTTGAGAGC	4	chr3	132797855	132797877	intron:Tbck
GGCACAGCTCACTCTTTACCTGT	4	chr3	30416935	30416957	intergenic:Gm10258-Mecom

GGCACAGGTCACGCTGGCACTGA	4	chr3	122209673	122209695	intergenic:Abca4-Gm4609
GGCCCAGGTCTCTCCTTCCGTGT	4	chr3	65995083	65995105	intergenic:Ccnl1-Veph1
GCCACAGCTCACTGCTTCAGTGA	4	chr3	100705297	100705319	intergenic:Man1a2-Gm23465
GGAACAGTTCTCTGCTTCACGGA	4	chr3	32741468	32741490	intergenic:Mrpl47/Ndufb5-Ndufb5
GTGACAGGTCACTGCTTCTCTGA	4	chr3	67087423	67087445	intron:Rsrc1
GGCAGAGCTCACTGCCTCACTGG	4	chr3	123682195	123682217	intron:Ndst3
TGAACAGGTCACTGCTTTACTGT	4	chr3	24469235	24469257	intergenic:Gm7536-Gm24704
GTCACAGCACACTTCTTCACGGG	4	chr4	133759780	133759802	intergenic:Arid1a-Gm25270
GGCTCTGGGTACTCCTTCACTGG	4	chr4	104427691	104427713	intron:Dab1
GGCAGGGGTAACTCCCTCACTGG	4	chr4	128777461	128777483	intron:Zfp362
GGCTCACCTCACACCTTCACGGG	4	chr4	128489529	128489551	intron:Csmd2
GGCACAGGGCCCACCCTCACGGG	4	chr4	104948842	104948864	intron:1700024P16Rik
GACACACTTCACTCCTTCATTGG	4	chr4	110903602	110903624	intron:Agbl4
GGCCCAGGTCACTCCTATTCAGG	4	chr4	112029629	112029651	intergenic:Skint1-Skint4
GGCACAGCTCGCTCCTTTCCAGG	4	chr4	154329598	154329620	intron:Prdm16
GGCACAGACCACTCCAACACAGA	4	chr4	132203788	132203810	intron:Ythdf2
GGCCCAGGTGAAGCCTTCACTGG	4	chr4	136659476	136659498	intron:Ephb2
GGCACAGCACACACCATCACAGA	4	chr4	41658924	41658946	exon:Cntfr
GGCAAAGGTCACTTTCTCACTGG	4	chr4	58498874	58498896	intron:Lpar1
GGCACAGGTCAGACATTCCCTGG	4	chr4	35104691	35104713	intergenic:Mob3b-Ifnk
GGCACTGGTCCCTCCTCCAGAGG	4	chr4	125381613	125381635	intergenic:1700041M05Rik-Grik3
GTCACAGGTCAGTCCATCTCTGA	4	chr4	67321873	67321895	intergenic:Gm11403-Hmgb1-rs18
GGTTGAGGTCACTACTTCACAGA	4	chr4	36389623	36389645	intron:Lingo2
GGAACAAGTCATTCCTTCAAGGC	4	chr4	14499312	14499334	intergenic:Gm11836-Slc26a7
GGAACAGGTGTCTCCTCCACTGA	4	chr4	115973408	115973430	intron:Faah
GGCATAGTTCTCTCCTACACAGA	4	chr4	127596196	127596218	intergenic:A630031M04Rik-1700112K13Rik
GGCACACATCCCTTCTTCACAGC	4	chr4	48118982	48119004	intergenic:Nr4a3-Stx17
GTCTAAGGTCACTTCTTCACAGC	4	chr4	56073650	56073672	intergenic:Gm12519-Gm12520
CGCACACGTCACTCCCTCTCCGT	4	chr4	84701046	84701068	intergenic:Gm12420-Gm12415
GGCTCAGCTGACTCCTTCCCAGA	4	chr4	48140738	48140760	intron:Stx17
GACACAGGTCATCCATTCACTGC	4	chr4	73052825	73052847	intergenic:Gm11251-Gm25769
ACCACAGGTCATTCCTTCATCGT	4	chr4	45916997	45917019	intron:E230008N13Rik
GACACTGGTCACTTCTTCCCAGT	4	chr4	96161978	96162000	intergenic:Gm12674-Gm12673
GAGACCGGTCAGTCCTTCACTGT	4	chr4	65534992	65535014	intron:Astn2
TGCACTGCTCACTCCTGCACTGC	4	chr4	9235211	9235233	intergenic:Gm23423-Gm26548
GGCACATTTCTGTCCTTCACAGC	4	chr4	155780294	155780316	intergenic:Gm13644-Tmem88b/Gm13644
GGCACAGGTCCCGACTTCGCAGA	4	chr4	149263993	149264015	intron:Kif1b
GGCACACGTCACCCAGTCACAGC	4	chr4	132786299	132786321	intron:Themis2
GGCAAAGGGCACTCTGTCACTGA	4	chr4	114402153	114402175	intergenic:Skint11-Trabd2b
GGCACAGGCCTTTCCTTCCCAGT	4	chr4	67142046	67142068	intergenic:Tlr4-Gm11403
GGCATAGGTCACTCTACCACAGT	4	chr4	55999808	55999830	intergenic:Gm12519-Gm12520
GGCACAGCTCACTCTTCCTCAGC	4	chr4	56459931	56459953	intergenic:Gm12518-Gm26144

GACACAGGTCATTCATGCACTGC	4	chr4	14522429	14522451	intron:Slc26a7
GTCACAGGTCCCTCCTGCCCTGC	4	chr4	143457073	143457095	intergenic:Oog4-Gm13042
GGCACAGAGCACTGCCTCACAGT	4	chr4	118624564	118624586	intron:Ebna1bp2
TGCACAGCTCACTGCTCCACAGC	4	chr4	84867739	84867761	intergenic:Gm12415-Cntln
GGCACAGCTCACTGCCCCACAGG	4	chr4	53236184	53236206	intergenic:4930412L05Rik-4930522O17Rik
AGCACAGTTCCCTGCTTCACTGT	4	chr4	153937622	153937644	intergenic:Gm13115-BC039966
GGCACAGGGCACAGCTACACAGC	4	chr4	141353095	141353117	intergenic:Gm13074-Gm13076
GGCTGAGGTCACTGCTTCTCAGG	4	chr4	119096617	119096639	intergenic:Gm12866-Slc2a1
GTCACAGCTCACAGCTTCACTGT	4	chr4	123248192	123248214	exon:Heyl
GAAACAAGTCACTCCTTCAAGGG	4	chr5	53009453	53009475	intergenic:Gm23532-Slc34a2
TGCACAGCTCACTACTCCACAGG	4	chr5	132588269	132588291	intergenic:Auts2-Gm25722
GGCACACTACACTCCTTCATGGG	4	chr5	17508188	17508210	intergenic:Speer4f-Sema3c
GTCACAGCTCAGTCCTTCTCTGG	4	chr5	25061103	25061125	intron:Prkag2
GGCACAGGCCGCCCCTGCACTGG	4	chr5	30670246	30670268	intron:Cenpa
TGATCTGGTCACTCCTTCACGGA	4	chr5	130820601	130820623	intergenic:Gm23761-Caln1
GGCCCAGGGCTCTCCTTCCCTGG	4	chr5	108704678	108704700	exon:Fgfrl1
GGCAGAGGTCACACATTGACTGG	4	chr5	47477370	47477392	intergenic:Gm7931-Slit2
GTCACTGGTCCCTCGTTCACAGG	4	chr5	77281341	77281363	exon:Rest
GGCACAGTTCACTCCTGGGCAGG	4	chr5	96087130	96087152	intron:Cnot6l
AGCACAGGGCAGTCCATCACTGT	4	chr5	37333425	37333447	exon:Evc
ACCACAGATCACTCCTTCATGGT	4	chr5	116775999	116776021	intergenic:Srrm4-4930569F06Rik
GGCACAGGCCACTCTTCCAGAGG	4	chr5	63505927	63505949	intergenic:Gm17384-3110047P20Rik/Gm9954
AGCACTGGTCACTCTTTCAGTGG	4	chr5	63475423	63475445	intergenic:Gm17384-3110047P20Rik/Gm9954
GGCACACGACACTCACTCACAGC	4	chr5	118758640	118758662	intron:Med13l
GACTCAGTTCTCTCCTTCACTGT	4	chr5	104360682	104360704	intergenic:Mepe-Spp1
GCCACATGTCACTCCTCCATGGT	4	chr5	32038470	32038492	intergenic:Gm17130-Bre
TGCACAGCTCACACCTGCACAGC	4	chr5	149757181	149757203	intron:B3galtl
TGCACAGCTCACACCTGCACAGC	4	chr5	149757286	149757308	intron:B3galtl
TGCACAGCTCACACCTGCACAGC	4	chr5	149757271	149757293	intron:B3galtl
TGCACAGCTCACACCTGCACAGT	4	chr5	149757316	149757338	intron:B3galtl
GACACAGGTCACTCATTTCCAGC	4	chr5	123355540	123355562	intron:Bcl7a
GGCAAAGGTCACTCATTGGCTGA	4	chr5	150939362	150939384	intergenic:Pds5b-Kl
TGCCCAGGTGACTCCTTGACTGC	4	chr5	121581046	121581068	intron:Aldh2
GCCACAGCTCACTCCTGCAGAGC	4	chr5	37276650	37276672	intron:Crmp1
GGCACAGGTTCCTCGTGCACTGC	4	chr5	144258603	144258625	intergenic:2900089D17Rik-Bri3/Baiap2l1
TGCACAGTTCACTGCCTCACAGA	4	chr5	28107237	28107259	intergenic:Insig1-En2
GCCACAGGTCACTGCCTCTCAGA	4	chr5	26939608	26939630	intergenic:Dpp6-Gm16057
GGCACAGCTCACTGGTCCACAGT	4	chr5	143893673	143893695	intron:Ankrd61
GCCTCAGGTCACTGCTTCTCTGA	4	chr5	126437155	126437177	intergenic:Gm23151-Gm24839
GGCACAAGGCAACCCTTCACCGG	4	chr6	32760267	32760289	intergenic:Gm13852-Chchd3
GGCACAGGACCCTCCTCCTCTGG	4	chr6	39548022	39548044	intergenic:8030453O22Rik-Dennd2a
AGCACAGGTCTCACCTCCACAGG	4	chr6	113384169	113384191	exon:Arpc4

GGCACAGGGAAGTCATTCACTGG	4	chr6	135385929	135385951	intergenic:Emp1-Gm25136
GGGACTTGTCACTCCTTCCCAGG	4	chr6	30617069	30617091	intron:Cpa5
TGCCCAGGTCAGTCCTTCCCAGG	4	chr6	52194956	52194978	intergenic:2700086A05Rik-Hoxa3
GGCACCAGGAACTCCTTCACAGA	4	chr6	4670725	4670747	intergenic:Casd1-Sgce
AGCACAGCTCACTCCCTTACAGA	4	chr6	91269826	91269848	intron:Fbln2
GGCAGAAGTCACACCTTCTCTGA	4	chr6	53619401	53619423	intron:Creb5
GTCTCAGCTCACTCCTTCTCTGA	4	chr6	25078626	25078648	intergenic:Tmem229a-Gm24443
GGGACAGATCTCACCTTCACAGA	4	chr6	126009954	126009976	intron:Ano2
GGCAAAGGACTCTCCTTCAAGGA	4	chr6	27931081	27931103	intergenic:Grm8-Grm8/Mir592
CGCACAGGGCATTCCTTCCCAGA	4	chr6	54062488	54062510	intron:Chn2
GACACAGTTCTCACCTTCACTGC	4	chr6	88446055	88446077	intergenic:Gm25178-Eefsec
GGCACAGGTCCCTCATTGCCTGG	4	chr6	142265561	142265583	intron:Slco1a5
GGCACAATTCACGTCTTCACTGT	4	chr6	65005527	65005549	intergenic:Gm15534-Smarcad1
GTCACAGGTCATTCCTTTGCTGT	4	chr6	117327009	117327031	intergenic:Rpl28-ps4-Gm9946
GGCACAGATTTCTCCTGCACAGT	4	chr6	145414035	145414057	intron:Ifltd1
GGCACAGGCCAGTCCTGCCCCGC	4	chr6	115515867	115515889	intergenic:Pparg-Tsen2
GTCCCAGGTCACTCTATCACTGT	4	chr6	126444087	126444109	intergenic:Ntf3-Kcna5
TGCAGAGCTCACTGCTTCACTGA	4	chr6	48537242	48537264	exon:1700026J14Rik
CTCACAAGTCACTGCTTCACAGA	4	chr6	130019553	130019575	intron:Klra6
AGCACAGTTCACTGCTCCACAGC	4	chr6	134902119	134902141	intergenic:Gpr19-Cdkn1b
GGCTCAGGTCACACCTTTTCTGG	4	chr7	86435702	86435724	intergenic:Olfr301-Olfr300-ps1
AGCTCAGGTCACTCCGCCACGGG	4	chr7	43677167	43677189	exon:Ctu1
AGCACTGGACACTCCTTCAGCGG	4	chr7	120997722	120997744	intron:4933427G17Rik
GGCAGAGGCCTCTCCTTGACTGG	4	chr7	66420068	66420090	intron:Aldh1a3
AGCACAGGAGACTCTTTCACTGG	4	chr7	119486851	119486873	exon:Pdilt
GCTACAGGACACACCTTCACTGA	4	chr7	145262064	145262086	intron:Tpcn2
TCCACAGGTCACTACTTCAGTGG	4	chr7	102240297	102240319	exon:Rhog
GGCACTGATCACTCCCTCGCAGA	4	chr7	65737148	65737170	intergenic:Tm2d3-Gm7551
GCCAGAAGCCACTCCTTCACAGC	4	chr7	137127960	137127982	exon:Mgmt
GGCACAGGTAAGAACTTCACAGA	4	chr7	71034511	71034533	intergenic:Gm24880-Gm10172
GGCACAGATCACATCTTCTCTGT	4	chr7	71433582	71433604	intergenic:Gm10295-Mctp2
GGGACAGGCCATCCCTTCACAGC	4	chr7	116278643	116278665	intron:Plekha7
GGCACACCTCACACCTTCCCAGA	4	chr7	96393262	96393284	intergenic:Tenm4-Rps11-ps5
ATCAAAGGTCACTTCTTCACAGT	4	chr7	79379962	79379984	exon:Rlbp1
AGCACTGGTCCCTCCCTCACTGC	4	chr7	80742494	80742516	intron:Iqgap1
GGCGCACGTGTCTCCTTCACAGA	4	chr7	101450748	101450770	intron:Pde2a
GACACAAGTCACTTCTTCAGGGA	4	chr7	135013326	135013348	intron:Dock1
GACAAAGTTCACTCCTGCACTGA	4	chr7	61031704	61031726	intergenic:Gm7367-A230006K03Rik
GGGACCGCTCACTCCTTTACAGT	4	chr7	78813100	78813122	intergenic:Mrps11-Det1
GGCAAAGGTCACCCCGGCACAGA	4	chr7	45261491	45261513	intron:Slc6a16
GGTAAAGGTCACTCCCTCATTGT	4	chr7	138232177	138232199	intron:Tcerg1l
GACACAGCTCACTCATTGACAGA	4	chr7	88310640	88310662	exon:Ctsc

GGCAGAGGTCATTCATTCTCAGC	4	chr7	49957761	49957783	intron:Slc6a5
GGCTCAGGTCACATCTGCACAGC	4	chr7	97738147	97738169	exon:Aqp11
CTCACAGGTCCCTCCTTCCCAGT	4	chr7	89994810	89994832	intergenic:Eed/Gm26529-Gm5341
GGCACAGGTGACTCTACCACTGT	4	chr7	19066837	19066859	intron:Rsph6a
GGCACAGGTCAGTCCAGGACAGC	4	chr7	73275874	73275896	intergenic:Gm5335-A730056A06Rik
GGCACAGTTCTCTCCTGCAGGGT	4	chr7	70185679	70185701	intergenic:Gm24120-Nr2f2
GGCACAGGGCTCTCTTTCAGTGC	4	chr7	64609722	64609744	intron:Apba2
GGCATAGGTCATTGTTTCACAGG	4	chr7	118737125	118737147	intergenic:Ccp110-9030624J02Rik
GGCCCAGGCCTCTGCTTCACTGC	4	chr7	38125448	38125470	intergenic:Ccne1-Gm22203
TGCACAGCTCACTGCTCCACAGA	4	chr7	125959310	125959332	intron:Gsg1l
AGCTCAGGACACACCTTCACTGG	4	chr8	12599592	12599614	intron:Spaca7
GGCAAAGTCCATTCCTTCACGGG	4	chr8	22740023	22740045	intergenic:Ikbkb-Plat
GGCACCCTTCACTCCTCCACTGG	4	chr8	97685352	97685374	intergenic:Gm7191-Gm23494
GGCACAGATCACATCTTCAGCGG	4	chr8	23954127	23954149	intron:Zmat4
GGCAGAAGTCACCACTTCACAGA	4	chr8	126749422	126749444	intergenic:Irf2bp2-Tomm20
GTCACATGTCATTCCTACACTGA	4	chr8	121667207	121667229	intergenic:Gm23299-Jph3
GGAACAAGCCCCTCCTTCACTGC	4	chr8	117366880	117366902	intron:Cmip
GGCACAGGCCACACCTACCCTGA	4	chr8	122549165	122549187	intergenic:Gm26497-Piezo1
GGCACACATCACTCCCTCGCAGC	4	chr8	14255940	14255962	intron:Dlgap2
GGCAGAGGGAGCTCCTTCACTGT	4	chr8	47656952	47656974	intergenic:Gm8623-Ing2
GGCAGAGGTCAGTCCATCTCAGC	4	chr8	110957401	110957423	exon:St3gal2
GACACAGCTGACTTCTTCACTGT	4	chr8	87812563	87812585	intron:Zfp423
TGCACATGTCTCTCCTCCACTGT	4	chr8	89547029	89547051	intergenic:Gm5356-Gm26331
GGCACAGGGGACTTCTGCACGGA	4	chr8	122216131	122216153	intergenic:Banp-Gm22
TGCACAGGGCCCTCCTTCCCTGT	4	chr8	103362615	103362637	intergenic:Gm8730-Cdh5
AGCACAGGACACTCCTCCAGGGC	4	chr8	111361142	111361164	intron:Fa2h
GGCACAGTTCACTTCTTCCAAGT	4	chr8	74731708	74731730	intron:Gm11033
GGCACTGCGCACTCGTTCACCGC	4	chr8	70897377	70897399	exon:Rpl18a
GGGACATGTCACTCTTACACTGC	4	chr8	93378199	93378221	intergenic:Ces1h-Ces5a
GGCACATGTCACTCTTACAGAGA	4	chr8	128189317	128189339	intergenic:Pard3-mmu-mir-21c
GGCACTGTTCACTGCTCCACAGG	4	chr8	34313677	34313699	intergenic:Gm6100-Gm8254
GGCTCAGGTAGCTGCTTCACTGC	4	chr8	47711587	47711609	exon:Cdkn2aip
GGCATGGGTAACTGCTTCACTGG	4	chr8	91762824	91762846	intergenic:Fto-Irx3
GGCTCAGTTCACACCCTCACTGG	4	chr9	35005365	35005387	intron:Kirrel3
GACACAGGATACTCCTTCCCTGG	4	chr9	118351506	118351528	intergenic:Gm22198-Gm26975
GGGACAGGTCAGTCCTCTACAGG	4	chr9	48499793	48499815	intergenic:Gm5617-Gm23653
TGCATAGGCCAATCCTTCACAGC	4	chr9	30941430	30941452	intergenic:Adamts15-Adamts8
AGCACAGGTCATTCCACCACAGC	4	chr9	36295587	36295609	intergenic:Gm3867-Gm7257
GTCACCGCTAACTCCTTCACTGT	4	chr9	99509850	99509872	intron:Armc8
GACACAGCTGCCTCCTTCACTGC	4	chr9	60096371	60096393	intron:Thsd4
GGCTAAGGTGACTTCTTCACTGT	4	chr9	93050845	93050867	intergenic:Plod2-Gm9621
GGCAGAGGTGCCTCCTTCCCAGA	4	chr9	64674338	64674360	intron:Megf11

GGTACAGGTCTCTCCCACACTGT	4	chr9	108951622	108951644	intergenic:Uqcrc1-Col7a1
GGCACAGGGAAGTCCTTCATGGT	4	chr9	110238541	110238563	exon:Smarcc1
GGCAGAGCTCACTACTACACGGT	4	chr9	75645811	75645833	intron:Scg3
GGCCCAGGCCACTTATTCACAGT	4	chr9	58890658	58890680	intron:Neo1
GGCAACGGTCTCTCCTTTACTGT	4	chr9	85567236	85567258	intergenic:Gm25125-Ibtk
GGCAGAGAACACTCTTTCACAGT	4	chr9	79549066	79549088	intergenic:Gm8116-Col12a1
GGCACAGGGCCCACCTGCACTGC	4	chr9	46080788	46080810	intron:Sik3
GGCACAGGTTACATGTTCACTGT	4	chr9	107824959	107824981	intron:Rbm6
GGCCCAGCTCACTCTCTCACAGC	4	chr9	47226988	47227010	intergenic:Gm4791-Gm22286
GGGCCAGGTCACTCCTTGGCTGT	4	chr9	54333977	54333999	intron:Gldn
GGCACAAGTCCCTCCTGGACAGC	4	chr9	117908367	117908389	intron:Zcwpw2
GGCCCAGGACACTCTTTCAGAGT	4	chr9	89280074	89280096	intergenic:Mthfs-AF529169
GGCACAGTCCACTGCTCCACAGA	4	chr9	102078980	102079002	intergenic:Ephb1-n-R5s88
GCCACAGATCCCTGCTTCACAGG	4	chr9	28652569	28652591	intron:Opcml
GGCCCAGGTCACTGCCTTACAGA	4	chr9	43765291	43765313	intron:Pvrl1
TGCAGAGGTGACTTCTTCACTGG	4	chr10	7065514	7065536	intergenic:Ipcef1-Cnksr3
GGGAATGGTCACTACTTCACAGG	4	chr10	3519551	3519573	intergenic:Ppp1r14c-Iyd
TGCACAGTTCACTCCAGCACAGG	4	chr10	54455913	54455935	intergenic:Gm26177-Gm25602
GGCACAGGCCACTCCAAGACAGG	4	chr10	9610754	9610776	intergenic:Gm9930-Samd5
AGGACAGTTCACTCCTTGACTGG	4	chr10	66645752	66645774	intergenic:Gm22594-1110002J07Rik
GGCAAAGGCCACACCTTAACAGA	4	chr10	84980636	84980658	intron:Ric8b
TGCAGAGGTCACACCTTCATTGA	4	chr10	73686839	73686861	intergenic:Gm15397-Gm6407
AGCACAAGTCACTTCTCCACAGT	4	chr10	123051521	123051543	intron:Mon2
GGCAAAGGTCACTACTTCTGGGG	4	chr10	84424562	84424584	intron:Nuak1
AGAAGAGGTCACTCCTCCACTGT	4	chr10	19352239	19352261	intergenic:Tnfaip3-Olig3
GGCTCAGCTCATTCCTTCCCCGA	4	chr10	128517483	128517505	intron:Esyt1
GCCACTGGTGACACCTTCACAGC	4	chr10	92778657	92778679	intron:Gm872
GACACAGCTCAAGCCTTCACTGC	4	chr10	50378669	50378691	intergenic:Grik2-Ascc3
GGCACAGGTACCCACTTCACGGC	4	chr10	128281764	128281786	intron:Stat2
GGCACCTGCCACTCCTACACGGT	4	chr10	42535711	42535733	intergenic:Snx3-Nr2e1
GGCACAGATAAATCCTTGACTGC	4	chr10	80042613	80042635	intergenic:Polr2e-Gpx4
CGCAGAGGTCACTCTTTCAGAGG	4	chr10	114074743	114074765	intergenic:Gm22507-Trhde
GGCAAATGTCACTCCATCAGAGA	4	chr10	38411471	38411493	intergenic:Gm24297-Gm22911
GGCACAGGACTCCCCTTCATGGT	4	chr10	73693509	73693531	intergenic:Gm6407-Pcdh15
GGCACAGCTCACTACCTCATAGC	4	chr10	80221137	80221159	intergenic:Efna2-Mum1
GGAACAGGTCACTCTTGGACAGG	4	chr10	126183820	126183842	intergenic:Gm22183-mmu-mir-378d
GGGAGAGGTCCCTCCTTGACAGC	4	chr10	95509675	95509697	intergenic:Mrpl42-Ube2n
AGCACCGGTCACTCCTTGCCTGC	4	chr10	8891442	8891464	intergenic:Gm26674/Sash1-Gm9930
GGCATAGCTCACTCATTCAGGGT	4	chr10	7444472	7444494	intergenic:Cnksr3-Ulbp1
AGCACAGGTTACTCTTTCAGAGT	4	chr10	5837897	5837919	intron:Rgs17
GACACAGGGCACTGCCTCACTGA	4	chr10	125320755	125320777	intron:Slc16a7
TGCACAGGTCACGGCTTCCCAGT	4	chr10	39413002	39413024	intron:Fyn

GCTACAGGCCACTCCTTCATTGG	4	chr11	120696382	120696404	intergenic:Aspscr1/Gm17178-Aspscr1
GGCACAGGTCAGCCCCTCTCTGG	4	chr11	57954428	57954450	intergenic:Gm12245-Gm26025
AGCACAGGAAACTCCCTCACAGA	4	chr11	43564812	43564834	intergenic:Ccnjl-Gm12150/Ccnjl
AGCCCAGGTCACTCCTTGCCCGG	4	chr11	34119928	34119950	intergenic:4930469K13Rik-Gm12121
GGCACAGTTCAGCCCTTCAATGA	4	chr11	110420183	110420205	intron:Map2k6
GGCAGGTATCACTCCTTCACAGC	4	chr11	35432118	35432140	intergenic:Gm12122-Slit3
GGCAGTGTTAACTCCTTCACTGC	4	chr11	70018361	70018383	intron:Dlg4
TGCACAGGTCACTTGTTGACTGG	4	chr11	77995828	77995850	intron:Phf12
GACAGCGGTCACTCATTCACTGA	4	chr11	76215282	76215304	intergenic:Glod4/Gemin4/Fam57a-Gemin4
AGCACAGGACACTCTTTCAGAGG	4	chr11	96523711	96523733	intron:Skap1
GTCCCAGGTCACTCCTTTATTGA	4	chr11	7732033	7732055	intergenic:Gm11986-U7
AGCACTGGTCACTCTTTCAGTGG	4	chr11	51798636	51798658	intergenic:Sar1b-Phf15
GGGAAAGGACCCTCCTTCACTGC	4	chr11	109098687	109098709	intergenic:Gm24149-E030025P04Rik
GGCACAAGGCACACCTTCATGGT	4	chr11	33578531	33578553	intron:Gabrp
GGCACATGCCACTCTTTCAGTGG	4	chr11	55354595	55354617	intergenic:Fat2-Gm12234
GGCACTGGTCACTCTTCCAGAGG	4	chr11	53475035	53475057	intergenic:Gm9837-Sowaha/Gm9945
AGTACAGGTCACTACTGCACAGA	4	chr11	37385830	37385852	intergenic:Tenm2-Gm12128
GTCACAGGTCAATACTTCAAAGC	4	chr11	44474062	44474084	intergenic:Ublcp1-Gm12156
GGCACAGGTGACTCCCAAACTGC	4	chr11	108805536	108805558	intron:Cep112
GGCACAGGTTAGTCACTCACTGC	4	chr11	100536643	100536665	intergenic:Acly-Ttc25
GTCACAGGGCCTTCCTTCACAGT	4	chr11	118777117	118777139	intergenic:Rbfox3-Gm11750
GGAACTGGTCACCCCTTCAGGGC	4	chr11	59823988	59824010	exon:Cops3
GGCCAAGGTCACTCCCTCCCAGC	4	chr11	114453616	114453638	intergenic:4932435O22Rik-Gm11689
GGCACCGGCCCCTCCTTGACAGC	4	chr11	82564061	82564083	intergenic:Tmem132e-Gm24612
GGCACAGGTGCCTCCCTCCCTGC	4	chr11	94161233	94161255	intergenic:B230206L02Rik-Tob1
TGCACAGGTCACTCGCTCTCTGC	4	chr11	120053072	120053094	intergenic:Aatk-Azi1
GGCACAGTTCACTCCCTGCCTGC	4	chr11	78129828	78129850	intron:Fam222b
GGCACAGGGCAGTACTGCACAGC	4	chr11	116663429	116663451	intron:Gm11744
CGCACAGGACACTCTTTCAGCGA	4	chr11	34125418	34125440	intergenic:4930469K13Rik-Gm12121
GGCACAGGTGGCTCTTTTACTGT	4	chr11	110547375	110547397	intergenic:Gm11682-Kcnj16
AGCACAGGTCAGTCTGTCACAGT	4	chr11	45631700	45631722	intergenic:Gm12162-Gm22284
AGCACAGGTCAGTCTGTCACAGT	4	chr11	45631588	45631610	intergenic:Gm12162-Gm22284
AGCACAGGTCAGTCTGTCACAGT	4	chr11	45631250	45631272	intergenic:Gm12162-Gm22284
AGCACAGGTCAGTCTGTCACAGT	4	chr11	45631398	45631420	intergenic:Gm12162-Gm22284
AGCACAGGTCAGTCTGTCACAGT	4	chr11	45631626	45631648	intergenic:Gm12162-Gm22284
AGCACAGGTCAGTCTGTCACAGT	4	chr11	45631436	45631458	intergenic:Gm12162-Gm22284
AGCACAGGTCAGTCTGTCACAGT	4	chr11	45631814	45631836	intergenic:Gm12162-Gm22284
AGCACAGGTCAGTCTGTCACAGT	4	chr11	45631324	45631346	intergenic:Gm12162-Gm22284
AGCACAGGTCAGTCTGTCACAGT	4	chr11	45631550	45631572	intergenic:Gm12162-Gm22284
GGGACAGGTCTCTCCTGCGCTGT	4	chr11	34261361	34261383	intron:Dock2
GGCACAGGTGACTCCGGCAGAGT	4	chr11	7624069	7624091	intergenic:Gm11986-U7
AGCACTGGTAACTGCTTCACAGA	4	chr11	20929328	20929350	intergenic:Gm22807-Gm23681

GGCACAGGTGACAGCTTAACGGG	4	chr11	114216525	114216547	intergenic:1700092K14Rik-Gm11690
GACACAGTTCCCTCCTTCTCAGG	4	chr12	44414194	44414216	intron:Nrcam
GGTACATGTCACTCATTCTCAGG	4	chr12	80306106	80306128	intergenic:Gm20727-Dcaf5
GGCAAAGGTCACACCTGCAAGGG	4	chr12	72119981	72120003	intron:Ccdc175
GTCACAGATGACTCCTTCAGGGG	4	chr12	21148878	21148900	intergenic:Asap2-Gm21989/Asap2
GTGACAGGTCACTTGTTCACAGG	4	chr12	85617170	85617192	intron:Jdp2
TGCACAGGACAAACCTTCACTGC	4	chr12	118859633	118859655	intergenic:Sp8-Abcb5
AGCAAAGGTCACTACTTAACAGA	4	chr12	77178002	77178024	intergenic:Gm22696-Fut8
CACACATGTCACTTCTTCACTGC	4	chr12	52575700	52575722	intergenic:Arhgap5-Gm24859
GGAACAAGCCACTCCTTCATGGC	4	chr12	83282735	83282757	intron:Dpf3
GCCATAGTTCACTCCCTCACTGC	4	chr12	54291641	54291663	intergenic:Gm24692-Sptssa
GGCACAGCTCACTCCATAAAAGT	4	chr12	67104290	67104312	intergenic:MDGA2/Mdga2-Mdga2
GGCACAGTCCACACATTCACTGT	4	chr12	67066637	67066659	intergenic:MDGA2/Mdga2-Mdga2
GGTAAAGGTCACTCCATCCCTGT	4	chr12	91874732	91874754	intergenic:Sel1l-Rpl31-ps1
GGTAAAGGTGAGTCCTTCACAGT	4	chr12	93555951	93555973	intergenic:Gm21614-Gm23249
GGCATAGGTCACACATTTACTGT	4	chr12	111188263	111188285	intergenic:4930595D18Rik-Traf3
GGCAAAGGTCACTAATTCAAAGC	4	chr12	98406427	98406449	intergenic:Gpr65-Kcnk10
AGCCCAGGTCACTGCTTCAGAGA	4	chr12	87419376	87419398	intergenic:Sptlc2-Alkbh1
GGCACAGATAAATGCTTCACAGA	4	chr12	78092958	78092980	intergenic:Gm24070-Gm24994
GGCACAGGTCCCTGCCACACAGA	4	chr12	29373611	29373633	intergenic:Gm23342-Myt1l
TACACAGGGCACTCCGTCACAGG	4	chr13	70074233	70074255	intergenic:Gm6132-Gm26018
GTCACAGGCCCCTCCTCCACAGG	4	chr13	21874088	21874110	intergenic:Hist1h2br-Gm11278
GGCACAGGTCACTCCCCTTCAGG	4	chr13	101957874	101957896	intergenic:Pik3r1-Gm25808
GGGACAGCTCACTACTACACAGG	4	chr13	111633170	111633192	intron:Gm15286
GGCACTGGTCACTCATTTATGGG	4	chr13	55391925	55391947	intergenic:Rgs14-Slc34a1
GGGAGAAGTAACTCCTTCACAGA	4	chr13	114721528	114721550	intergenic:4930544M13Rik-Mocs2
GGCAAAGGTCTGTCATTCACTGG	4	chr13	116995252	116995274	intron:Parp8
GGCAGACTTCAGTCCTTCACTGA	4	chr13	117446498	117446520	intergenic:Gm6421-Hcn1
GGTACAGGACACTCCTTTCCTGA	4	chr13	49208102	49208124	exon:1110007C09Rik
GTCACAGATCCCTCCATCACTGC	4	chr13	38052934	38052956	exon:Riok1
GGCACAGATGAAACCTTCACAGT	4	chr13	41460734	41460756	intron:Nedd9
GGCACAGTACCTTCCTTCACAGC	4	chr13	18178837	18178859	intron:Pou6f2
CGCACAAGTCTCTCCTTCAATGT	4	chr13	97224851	97224873	intergenic:Hexb-Enc1
GACACAGGTGACTCAGTCACAGA	4	chr13	35040294	35040316	intergenic:Eci2-Gm22674
GGCACAGGGGGCTACTTCACAGT	4	chr13	113815784	113815806	intron:Arl15
GGGACGGGTCTCTCTTTCACAGA	4	chr13	102685569	102685591	intergenic:Gm25808-Cd180
GGCACATGTCCCTCCCTCCCAGT	4	chr13	99785560	99785582	intergenic:Gm24471-Cartpt
GGAACAGGTCACGCTGTCACTGA	4	chr13	47202805	47202827	intron:Rnf144b
TGCACAGCACACTGCTTCACCGT	4	chr13	59388294	59388316	intergenic:Ntrk2-Agtpbp1
GGCAGAGATCACGGCTTCACTGT	4	chr13	24980017	24980039	intron:Gpld1
GACACATGTCACTGCTTTACTGT	4	chr13	77357719	77357741	intron:2210408I21Rik
AGCACAGGTAACTGCTTCAAGGG	4	chr13	72120073	72120095	intergenic:Irx1-Rpl9-ps4

GGCAGAGGTTCCTGCTTCACTGG	4	chr13	18036840	18036862	intergenic:Vdac3-ps1-Pou6f2
GGCATAGAACATTCCTTCACAGG	4	chr14	18589827	18589849	intron:Gm20678
GGCAGAGGTCAGACCTTTACAGG	4	chr14	110667131	110667153	intergenic:Gm25670-Gm26255
GGGACAGGACACTCCACCACAGA	4	chr14	121460500	121460522	exon:Slc15a1
GGTACAGCTCACTCATTCCCAGG	4	chr14	77348523	77348545	intron:Enox1
GTCACATGTCCCTCCTTGACAGG	4	chr14	119159935	119159957	intron:Hs6st3
GGCCCAGGACACTTCTACACAGA	4	chr14	70345272	70345294	intron:Slc39a14
GGCACCCTTGACTCCTTCACTGA	4	chr14	30108264	30108286	intron:Cacna1d
AGCACAGGGCAGTCCCTCACAGC	4	chr14	103298785	103298807	exon:Mycbp2
GGCAGAGGACACTCCCTCTCTGT	4	chr14	88471032	88471054	exon:Pcdh20
GGCCCAGGACACGCCTTCTCTGA	4	chr14	77803680	77803702	intergenic:Gm1587-Dnajc15
GGGACTGGTCACTCCATCTCTGT	4	chr14	71899405	71899427	intergenic:Gm9192-Gm23735
GTCACAGGGCGCTCCTTCCCTGC	4	chr14	54975080	54975102	intron:Myh7
GTCACAGGGCGCTCCTTCCCTGC	4	chr14	54948460	54948482	intron:Myh6
GGCACAGGGAAGTCCTTCATGGT	4	chr14	84756745	84756767	intergenic:Pcdh17-Gm23926
GGTAAAGGTCACTCCATCAGGGC	4	chr14	103208823	103208845	exon:Mycbp2
GGCTGAGGTCACTCTTTCATTGT	4	chr14	113713695	113713717	intergenic:Gm4487-Gm24073
GGCCCTGGTCACTGCATCACAGT	4	chr14	23568960	23568982	intron:Kcnma1
CACACAGGTCACTGCTCCACAGC	4	chr14	78320088	78320110	intergenic:Gm26197-Akap11
GGTAATGGTCACTGCTTCACTGG	4	chr14	38679108	38679130	intergenic:Nrg3-4930529F24Rik
TGCACAGATCACTGCCTCACAGA	4	chr14	58756509	58756531	intergenic:Gm25614-Gm9022
GGCAAAGGTCACCGCTTCATGGC	4	chr14	79256899	79256921	intergenic:Zfp957-Rgcc
TACACAGGTCACTGCTTCCCAGC	4	chr14	41161225	41161247	intergenic:Mbl1-Sftpd
CGCCCTAGTCACTCCTTCACAGG	4	chr15	73596230	73596252	intron:Slc45a4
GCCACAGGACACTACTCCACTGG	4	chr15	16435428	16435450	intergenic:Gm25550-Cdh9
GCCACAGATAAATCCTTCACAGA	4	chr15	59069431	59069453	intron:Mtss1
GGAACCGGACACTTCTTCACGGC	4	chr15	59652800	59652822	exon:Trib1
GGCAGGGGACACTCCTTTACAGC	4	chr15	66161839	66161861	intron:Kcnq3
GGCACAGGTTAATCCTCCAAAGC	4	chr15	54559750	54559772	intergenic:Colec10-Mal2
GGCACCTGTCATTCCTACACTGA	4	chr15	42255116	42255138	intergenic:Gm24751-Angpt1
GGCACTGATCCCTCCTTCCCAGA	4	chr15	73040223	73040245	intron:Trappc9
GGCACAGGGCACTCCCTGAGGGG	4	chr15	100696113	100696135	exon:1810009N23Rik
GGCAAAGGTCCCTCACTCACCGA	4	chr15	89475488	89475510	exon:Arsa/C230037L18Rik
GGGACAGCTCTCTCCTTCATGGA	4	chr15	82925781	82925803	intergenic:Tcf20-Mir3080
GGAACGGGTCACTACTTCTCTGT	4	chr15	4840854	4840876	intergenic:C6-Mroh2b
GTCCCAGGTTACTCCTTCTCTGT	4	chr15	12598827	12598849	intergenic:Pdzd2-Gm24302
GGCCCTTGTCACTCCCTCACAGT	4	chr15	78172223	78172245	intron:Ift27
GGCACAGGGAAGTCCTTCGCTGT	4	chr15	81887077	81887099	intron:Aco2
GGCACATATCACTCTTACACTGA	4	chr15	52058612	52058634	intergenic:Aard-Slc30a8
AGCACTGGTCACTCCTGCATGGC	4	chr15	8820980	8821002	intergenic:Slc1a3-Gm5210
GGCTCAGGGCTCTCCTTCAGAGA	4	chr15	83022040	83022062	intron:Nfam1
GTCACAGGTCACTTGCTCACTGT	4	chr15	85020886	85020908	intron:Upk3a

GCCACAGGTCCCTCCTTCTGGGC	4	chr15	58618468	58618490	exon:Fer1l6
CCCACAGGTCACTGCTTCTCAGC	4	chr15	100389836	100389858	intron:Slc11a2
GGCACTGCTCAGTCATTCACAGG	4	chr16	52267986	52268008	intron:Alcam
GACTCAGGTCACTCTTTTACTGG	4	chr16	78347816	78347838	intron:Cxadr
GACACAGTTCAGTCCTCCACAGA	4	chr16	33181254	33181276	intergenic:Gm23916-Osbpl11
GGCACAGAACATTCATTCACAGA	4	chr16	13593974	13593996	intron:Parn
CTCTCAGGTCACTCCTTCATGGA	4	chr16	41873998	41874020	intron:Lsamp
GGTAAAGGACACACCTTCACAGC	4	chr16	49758308	49758330	intron:Ift57
AGCACTTGTCACTCCTACACAGC	4	chr16	41531939	41531961	intergenic:AC124193.1-Lsamp
AGCACAGTTAAGTCCTTCACAGT	4	chr16	29726716	29726738	intergenic:Opa1-Gm26834
TGCACAGATCCTTCCTTCACTGT	4	chr16	26166418	26166440	intergenic:Leprel1-Cldn1
AGCACTGGTCACTCTTTCAGGGG	4	chr16	89893388	89893410	intron:Tiam1
GGCACCTGCCACTCCTGCACTGC	4	chr16	10741536	10741558	intron:Clec16a
TGCACAGGTAACTCTTTCATTGT	4	chr16	70088906	70088928	intergenic:Speer2-Gbe1
GGGACAGGGCATTCCTTCAGAGC	4	chr16	92435157	92435179	intron:Rcan1
GACACATGTCACTCCTGCCCAGT	4	chr16	18307551	18307573	intron:Tango2
GTCACAGGTCTCTCCTTGATTGT	4	chr16	11330416	11330438	intron:Snx29
GGCACAGGGCACGCTGTCACTGT	4	chr16	23418209	23418231	intergenic:St6gal1-Gm23430
TGCACAGGTACCTGCTTCACAGT	4	chr16	90780192	90780214	exon:Urb1
GGCACCGGTCTGTGCTTCACTGG	4	chr16	38397533	38397555	intron:Pla1a
GGCACAGGGCACTGCCTCCCTGC	4	chr16	20738241	20738263	exon:Chrd
GTCACACGTCACTGATTCACTGA	4	chr16	28840448	28840470	intron:Mb21d2
GGCACAGTTCACTGCCCCACTGA	4	chr16	55277653	55277675	intron:Zpld1
GGTAGAGGCCACTCCTTCAGTGG	4	chr17	25068464	25068486	intron:Tmem204
GACACAGGTAACTCCTTTAAAGA	4	chr17	55618680	55618702	intron:Vmn2r118
GCCACAGGTCATTCCACCACTGC	4	chr17	17568389	17568411	exon:Lnpep
GCCACAGCTCCATCCTTCACAGC	4	chr17	45670826	45670848	intron:Tmem63b
AGCACGGGTCACTCTCTCACAGA	4	chr17	72690384	72690406	intergenic:Alk-Gm26963
AGGACAGGTGCCTCCTTCACAGC	4	chr17	26660313	26660335	intergenic:Ergic1-Atp6v0e
GGCAAAAGTCACTCGCTCACTGA	4	chr17	20053922	20053944	intergenic:Vmn2r104-Vmn2r-ps126
GGCAGAGGTCTCTCTCTCACAGA	4	chr17	81789361	81789383	intergenic:Slc8a1-Rpl31-ps25
GGGACAGATCACTCATTCCCTGC	4	chr17	53323339	53323361	intergenic:Kcnh8-Efhb
GGCACAAGGCACTCCTTGAGGGA	4	chr17	54978845	54978867	intergenic:Gm26291-Gm17377
AGCACAGCTCACTCTTTCAGGGT	4	chr17	33115560	33115582	intergenic:Zfp563-Morc2b
AGCACAGGTCACTGCTTCTGAGT	4	chr17	85653386	85653408	intergenic:Six3-Six2
TGCACAGGTCACTGTTACACGGT	4	chr17	61871279	61871301	intergenic:Gm27023-Gm25800
GCCACTGCTGACTCCTTCACGGG	4	chr18	64810722	64810744	intergenic:Gm24504-Nedd4l
GGCACATGGCACTCCTTCTATGG	4	chr18	81056685	81056707	intergenic:Gm25718-4930594M17Rik
GGCTCAAGTGACTCTTTCACGGG	4	chr18	53678265	53678287	intergenic:Prdm6-Cep120
GGAACAGGTCATGCCTTCTCCGA	4	chr18	13409584	13409606	intergenic:Gm22251-Zfp521
AGCACAGGTGACTCCATAACGGC	4	chr18	81816004	81816026	intergenic:4930594M17Rik-Gm26012
ATCAGAGGTCACTCCTTCAGTGA	4	chr18	13953640	13953662	intron:Zfp521

GGAACAGGTGACTCTTACACAGA	4	chr18	72643240	72643262	intergenic:Dcc-Gm25509
GGCACAGATCACTCTCTCCCTGA	4	chr18	77244934	77244956	intron:St8sia5
GGCCCAGGTCAGCCCTGCACAGC	4	chr18	77488775	77488797	intron:Rnf165
GGGACATGTCACTCCTACAGTGC	4	chr18	23545105	23545127	intron:Dtna
GGCTCAGGACACTCTTTCAGTGA	4	chr18	3356852	3356874	intergenic:Crem-Gm6225
GGCCCAGCTCACTCTTTCCCTGC	4	chr18	15266283	15266305	intergenic:Kctd1-Aqp4
TGCTCACGTCACTGCTTCACTGT	4	chr18	84455607	84455629	intron:Zfp407
AGCACAGGGCACTGCCTCACTGC	4	chr18	69478022	69478044	intron:Tcf4
GGCACTGGGCACTGCTTCCCAGC	4	chr18	34419547	34419569	intron:Pkd2l2
GTGACAGGTCACCTCTTCACTGG	4	chr19	42830808	42830830	intron:Hpse2
GGCACATGTCTTTCCCTCACTGG	4	chr19	6472673	6472695	intron:Nrxn2
GACACAGGTCACCCCTTTATTGA	4	chr19	6025008	6025030	intergenic:Gm10814-Gm8034
CCCTCTGGTCACTCCTTCACTGT	4	chr19	40919359	40919381	intergenic:Zfp518a-Blnk
TGCACAGGTCATTCCTTTCCTGT	4	chr19	14564397	14564419	exon:Tle4
GGCACAGGCCACGCATGCACAGA	4	chr19	26687048	26687070	intron:Smarca2
GGCAAAGGTTAATCCTTCAGGGT	4	chr19	34613140	34613162	exon:I830012O16Rik
GGCCCAGGTCTCTCTGTCACTGA	4	chr19	6489348	6489370	intron:Nrxn2
AGCACAGTTGACTGCTTCACAGG	4	chr19	41751938	41751960	intergenic:Gm22601-Arhgap19
GGCACAGCTGAATGCTTCACTGC	4	chr19	44753063	44753085	intergenic:Gm26644-Pax2
GGCACAGGACACAGCATCACAGG	4	chr19	54321878	54321900	intergenic:Adra2a-Gpam
GGCTCAGGTGACTCTTCCACAGG	4	chrX	12878710	12878732	intergenic:Gm25063-Gm14524
CCCATTGGTCACTCCTTCACTGC	4	chrX	77106605	77106627	intergenic:Gm14714-Gm5398
TGCACTAGTCTCTCCTTCACAGC	4	chrX	40014062	40014084	intergenic:Gm7190-Gm14643
GTCACAAGTCATTCTTTCACAGA	4	chrX	142237233	142237255	intron:Nxt2
GCCAAAGGTCACTCCTTTCCAGC	4	chrX	46291513	46291535	intron:4930515L19Rik
GGCACAGGTCATTTCTTACCTGC	4	chrX	165520553	165520575	intergenic:Glra2-Gm15236
GTAACAGGTCACTATTTCACTGA	4	chrX	74083216	74083238	intron:Mecp2
GGCACAGCTCACTCCAACAGAGC	4	chrX	46937273	46937295	intergenic:Gm7722-Gm14609
GGCAAAAGTCAGTGCTTCACAGG	4	chrX	72824422	72824444	intergenic:Gm14736-Gabrq

Supplementary Table 3. Nr6a1 on-target locus and examined off-target loci.

Target	Locus (GRCm38/mm10)	Target sequences
Nr6a1 target	Chr2: 38727819-38727841	GGCACAGGTCACTCCTTCACCGT
Off-target-1	Chr4: 66563148-66563170	GGAACAGGTCAGTCCTTCACAGT
Off-target-2	Chr17: 69304961-69304983	GGCAAAGGCCACTCCTTCACTGG
